# T‐2 Toxin Exploits Gut‐Derived *Staphylococcus Saprophyticus* to Disrupt Hepatic Macrophage Homeostasis

**DOI:** 10.1002/advs.202512828

**Published:** 2025-09-06

**Authors:** Yuanyuan Zhu, Liu Xu, Fangrui Guo, Jianyu Qu, Xiangyan Liu, Qiurong Xu, Jie Sheng, Jiangping Wang, Xiaohong Xie, Ruimin Ren, Chuan Zhou, Sisi Yan, Shuiping Liu, Zhihang Yuan, Rongfang Li, Jing Wu, Jine Yi, Yulong Yin, Lixin Wen, Ji Wang

**Affiliations:** ^1^ Hunan Engineering Research Center of Livestock and Poultry Health Care College of Veterinary Medicine Hunan Agricultural University Changsha 410128 China; ^2^ Changsha Lvye Biotechnology Co., Ltd. Changsha 410100 China; ^3^ Key Laboratory of Livestock and Poultry Resources (Pig) Evaluation and Utilization Ministry of Agriculture and Rural Affairs College of Animal Science and Technology Hunan Agricultural University Changsha 410128 China; ^4^ Yuelushan Laboratory Changsha 410128 China; ^5^ School of Basic Medicine School of Public Health Hengyang Medical School University of South China Hengyang 42100l China; ^6^ Institute of Subtropical Agriculture Chinese Academy of Sciences Changsha 410125 China; ^7^ Institute of Yunnan Circular Agriculture Industry Pu'er 665000 China; ^8^ Hunan Collaborative Innovation Center of Animal Production Safety Changsha 410128 China

**Keywords:** gut‐liver axis, macrophage homeostasis, NOD2, *Staphylococcus saprophyticus*, T‐2 toxin

## Abstract

T‐2 toxin, a mycotoxin that frequently causes hidden contamination in food and animal feed, poses a substantial threat to both human and animal health. *Staphylococcus saprophyticus* (*S. saprophyticus*) is an opportunistic pathogen that widely infects humans and various animals. However, the specific conditions under which it becomes pathogenic, as well as the mechanisms underlying its pathogenicity remain unknown. In this study, it is found that a sub‐cytotoxic dose of T‐2 toxin in piglet and mouse models promotes the proliferation of intestinal *S. saprophyticus* and facilitates its translocation to the liver. Subsequent mechanistic investigations reveal that the translocated bacterium activates the nucleotide‐binding oligomerization domain 2 (NOD2)‐microtubule‐associated protein 1 light chain 3 and NOD2‐C‐C motif chemokine ligand 2 signaling pathways in Kupffer cells (KCs), thereby provoking autophagy in KCs and recruiting monocytes to the liver, alongside the M1 polarization of hepatic macrophages. Furthermore, modulation of the intestinal microbiota by xylo‐oligosaccharides, as opposed to antibacterial agents, effectively mitigates the disruption of hepatic macrophage homeostasis. This work shows, for the first time, the pivotal role of *S. saprophyticus* in mycotoxin‐induced impairment of liver immune function. It reveals the interaction between opportunistic pathogens, environmental toxins, and immune homeostasis.

## Graphical abstract

T‐2 toxin promotes the proliferation of intestinal *S. saprophyticus* and facilitates its translocation to the liver in piglets and mice. The translocated bacteria trigger autophagy in Kupffer cells and recruit monocytes to the liver through the nucleotide‐binding oligomerization domain 2 (NOD2) signaling pathway, alongside the M1 polarization of hepatic macrophages. Ultimately, this disrupts the immune homeostasis of the liver.

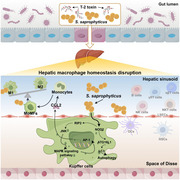

## 1 Introduction

Animals are exposed to significantly higher concentrations of pathogenic microorganisms through their diet or feed compared to humans. Upon breaching the intestinal barrier, these pathogens first migrate to the liver. Consequently, the liver serves as a crucial immune organ in animals, where hepatic macrophages, known as Kupffer cells (KCs), eliminate over 80% of invading pathogens. This process acts as a secondary defense mechanism to prevent further bacterial dissemination^[^ ^1^, ^2^ ^]^ The liver is maintaining balance between immune defense and metabolic homeostasis through its unique immune microenvironment (IME). The IME primarily consists of a network of immune cells, including KCs, liver sinusoidal endothelial cells, natural killer cells, and natural killer T cells.^[^ ^3^ ^]^ The homeostasis of hepatic macrophages is critical for preserving liver immune function, metabolic equilibrium, and tissue repair. Central to this homeostasis are factors such as the stability of macrophage cell numbers, functional polarization across various states, and their spatial localization within the liver.^[^ ^4^, ^5^ ^]^ Disruption of this homeostatic balance can impair pathogen control, facilitating the translocation of microorganisms into the systemic circulation via the vena cava, which can lead to multiorgan infections or potentially life‐threatening sepsis.^[^ ^6^ ^]^

*Staphylococcus saprophyticus* (*S. saprophyticus*) is a zoonotic Gram‐positive coccus that presents potential health risks to both humans and animals. It is a leading pathogen responsible for community‐acquired urinary tract infections, particularly affecting young women.^[^ ^7^ ^]^ Furthermore, it has been established as an opportunistic pathogen in various animals, including pigs,^[^ ^8^ ^]^ ducks.^[^ ^9^ ^]^ cattle,^[^ ^10^ ^]^ goats, and sheep.^[^ ^11^ ^]^ The molecular epidemiological characteristics of *S. saprophyticus* suggest that its global dissemination is closely associated with factors such as the antibiotic resistance genes it harbors, phage platelet‐binding proteins, and its biofilm‐forming capabilities.^[^ ^12^ ^]^ However, previous research has predominantly concentrated on this bacterium's drug resistance and its detrimental effects on the urinary and reproductive tracts. To date, the factors driving *S. saprophyticus* proliferation and pathogenicity in animal hosts remain uncharacterized.

T‐2 toxin, a trichothecene mycotoxin produced by *Fusarium* species, predominantly contaminates grains such as wheat, corn, and barley, along with their derivative products, as well as nuts, cereals, fruits and herbs.^[^ ^13^ ^]^ Current global warming and climate change are making crops such as corn and wheat more susceptible to fungal colonization and mycotoxin contamination.^[^ ^14^ ^]^ According to a mycotoxin contamination survey encompassing 78 countries, the contamination rate of T‐2 toxin in pig feed is 33%, while in poultry feed it is 35%.^[^ ^15^ ^]^ Due to its chemical stability, T‐2 toxin is difficult to be completely removed from food and feed, posing a great threat to human and animal health. T‐2 toxin has been demonstrated to disrupt the gut microbiota and intestinal barrier in animals.^[^ ^14^ ^]^ In our prior work, we observed that a sub‐cytotoxic dose of T‐2 toxin promoted the abundance of *S. saprophyticus* in the intestine and the liver, accompanied by alterations in immune homeostasis within the liver. Nevertheless, the interactions between these changes and their underlying mechanisms remain unclear. This study aims to elucidate the interactions between *S. saprophyticus* and the mycotoxin T‐2, alongside their impact on hepatic immune homeostasis in cross‐species models utilizing piglets and mice, with an additional focus on exploring potential intervention strategies.

## 2 Results

### 2.1 T‐2 Toxin Induced Imbalance of Immune Microenvironment in the Liver of Piglets and is Associated with *Staphylococcal* Infection

**Figure 1:**
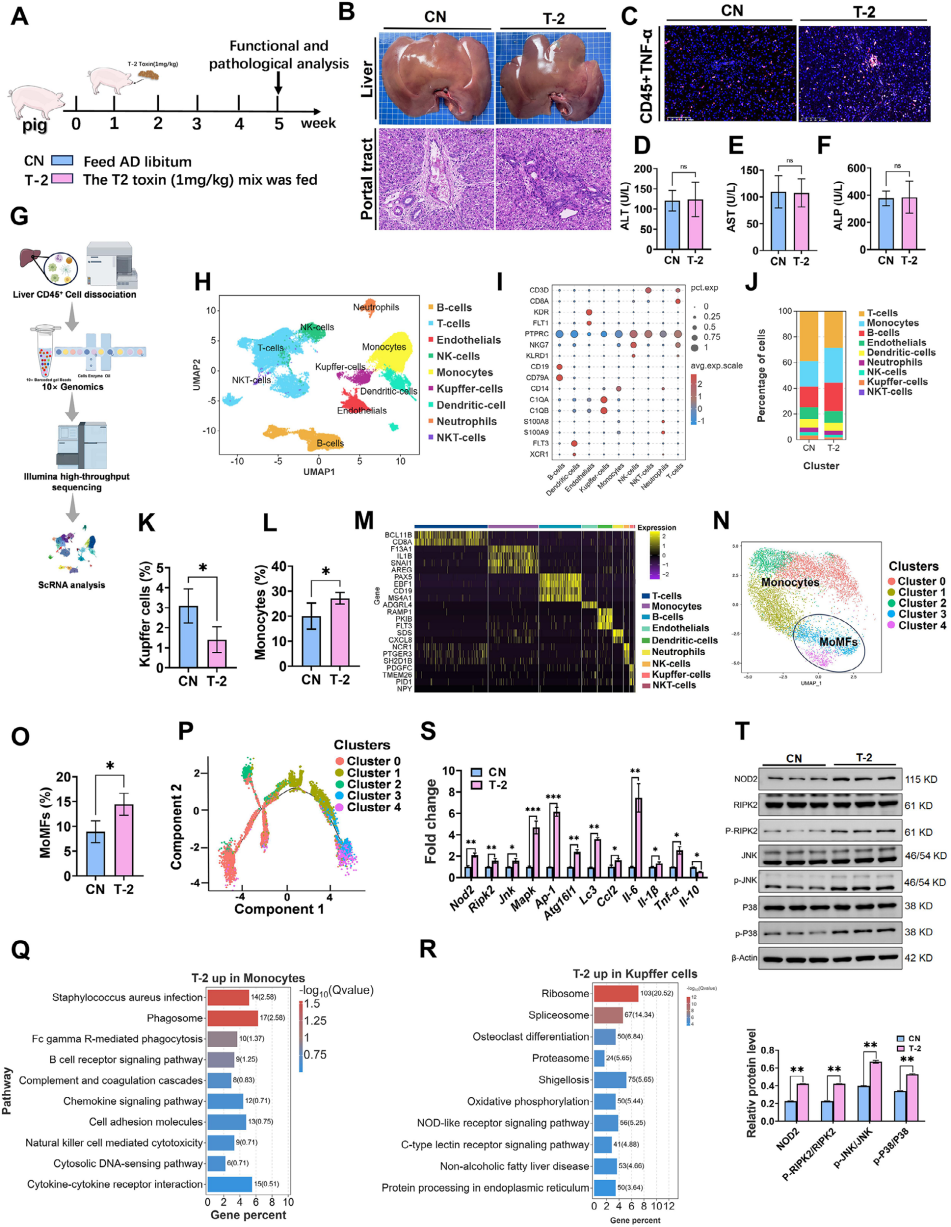
T‐2 toxin disrupts liver immune function. A) Experimental scheme of T‐2 toxin exposure in piglets. (*n*=8 per group). B) Gross observation and H&E staining of the liver. Scale bars, 100 µm. C) Immunofluorescence double staining of CD45 and TNF‐α. Scale bars, 100 µm. D–F) Serum biochemical parameters of ALT (D), AST (E), and ALP (F). G) Flow chart of scRNA‐seq. H) CD45^+^ cell subpopulation classification UMAP plot in liver. I) Bubble map of cellular subpopulation marker gene. J) Subpopulation cell frequency plot. K,L) Subpopulation proportion change plot of Kupffer cells (K) and monocytes (L). M) Heatmap of cellular subpopulation differential gene. N) Cellular subpopulation distribution along differentiation trajectory. O) MoMFs subpopulation proportion change plot. P) Cellular subpopulation differentiation trajectory plot. Q,R) Monocytes (Q) and Kupffer cells (R) upregulated genes KEGG pathway enrichment analysis in T‐2 group. S) Inflammatory factors, NOD2 pathway and autophagy‐related genes expression levels. T) Proteins expressions levels of NOD2 pathway. Data represent mean ± SD. *p < 0.05, **p < 0.01, ***p < 0.001, ****p < 0.0001; ns, no significance.

First, we investigated the impact of T‐2 toxin on liver injury in piglets. The piglets were fed either a standard diet (CN) or a diet containing 1 mg kg^−1^ of T‐2 toxin (T‐2), which they could eat ad libitum (**Figure** **1**A). Initially, we observed significant inflammatory cell infiltration in the liver portal tract (Figure 1B). Immunofluorescence analysis revealed that exposure to T‐2 toxin significantly increased the fluorescence intensity of both protein tyrosine phosphatase receptor type C (CD45) and tumor necrosis factor (TNF) ‐α in the tissues (Figure 1C). Concurrently, the levels of the pro‐inflammatory factors *Il‐6*, *Il‐1β*, and *Tnf‐α* were significantly elevated, indicating hepatic inflammation (Figure 1S).

No significant differences were observed in liver injury indicators, including Alanine transaminase (ALT), Aspartate aminotransferase (AST), and alkaline phosphatase (ALP), indicating that hepatocytes were not damaged (Figure 1D–F). We hypothesized that nonparenchymal liver cells might be involved in the inflammatory response. To test this hypothesis, we used flow cytometry to isolate CD45+ immune cells from piglet livers and performed single‐cell RNA sequencing (scRNA‐seq), followed by visualization of marker gene expression patterns across different cell subpopulations (Figure 1G–J). The results showed that T‐2 toxin altered the composition of liver immune cells: resident Kupffer cells (KCs) were significantly reduced, while monocytes were significantly increased (Figure 1K,L). Although changes were observed in other immune cell populations, they were not statistically significant (Figure S1A–G, Supporting Information).

Further subclustering of monocytes revealed that they could be divided into monocytes and monocyte‐derived macrophages (MoMFs) (Figure 1M–O). Pseudotime trajectory analysis showed that cluster 0 macrophages differentiated into MoMFs in various states (Cluster 3 and 4), which were predominantly distributed in the T‐2 group (Figure 1P; Figure S1H,I, Supporting Information). This suggested that MoMFs may exacerbate liver inflammation by activating upregulated genes in monocytes and KCs exceeded that of downregulated genes following T‐2 treatment (Figure S1J, Supporting Information). kyoto encyclopedia of genes and genomes (KEGG) enrichment analysis revealed that these upregulated genes were significantly associated with *Staphylococcus aureus* infection and the nucleotide‐binding oligomerization domain (NOD)‐like receptor signaling pathway (Figure 1Q,R). CellChat analysis of intercellular communication networks revealed strong interactions between KCs and monocytes, T cells, and B cells. Specifically, the communication between KCs and monocytes was potentially mediated by the C‐C motif chemokine ligand (CCL) pathway and the Galectin‐9‐CD45 ligand‐receptor pair (Figure S1K,M, Supporting Information). Finally, we examined the expression levels of cytokines and pathway‐related genes in liver tissues. The results showed that pro‐inflammatory factors, including Interleukin‐6 (*Il‐6*), *Il‐1β*, and *Tnf‐α;* chemokines (*Ccl2*); NOD2 pathway‐related genes including *Nod2*, receptor‐interacting serine/threonine‐protein kinase 2 (*Ripk2*), c‐Jun N‐terminal kinase(*Jnk*), mitogen‐activated protein kinase (*Mapk*), and activator protein 1(*Ap‐1*); and autophagy‐related genes, including autophagy‐related protein 16‐like 1 (*Atg16l1*), microtubule‐associated proteins 1A/1B light chain 3 (*Lc3*), were significantly increased, while anti‐inflammatory factors (*Il‐10*) was significantly decreased in the T‐2 group (Figure 1S). Additionally, NOD2 pathway‐related proteins (NOD2, RIPK2, JNK, P38) were activated (Figure 1T). These findings preliminarily suggest that *Staphylococcus* may be the driving force behind the imbalance of the liver immune microenvironment, thereby inducing liver inflammation.

### 2.2 T‐2 Toxin‐Induced Disruption of the Intestinal Barrier Leads to Viable Bacterial Colonization in the Liver

**Figure 2:**
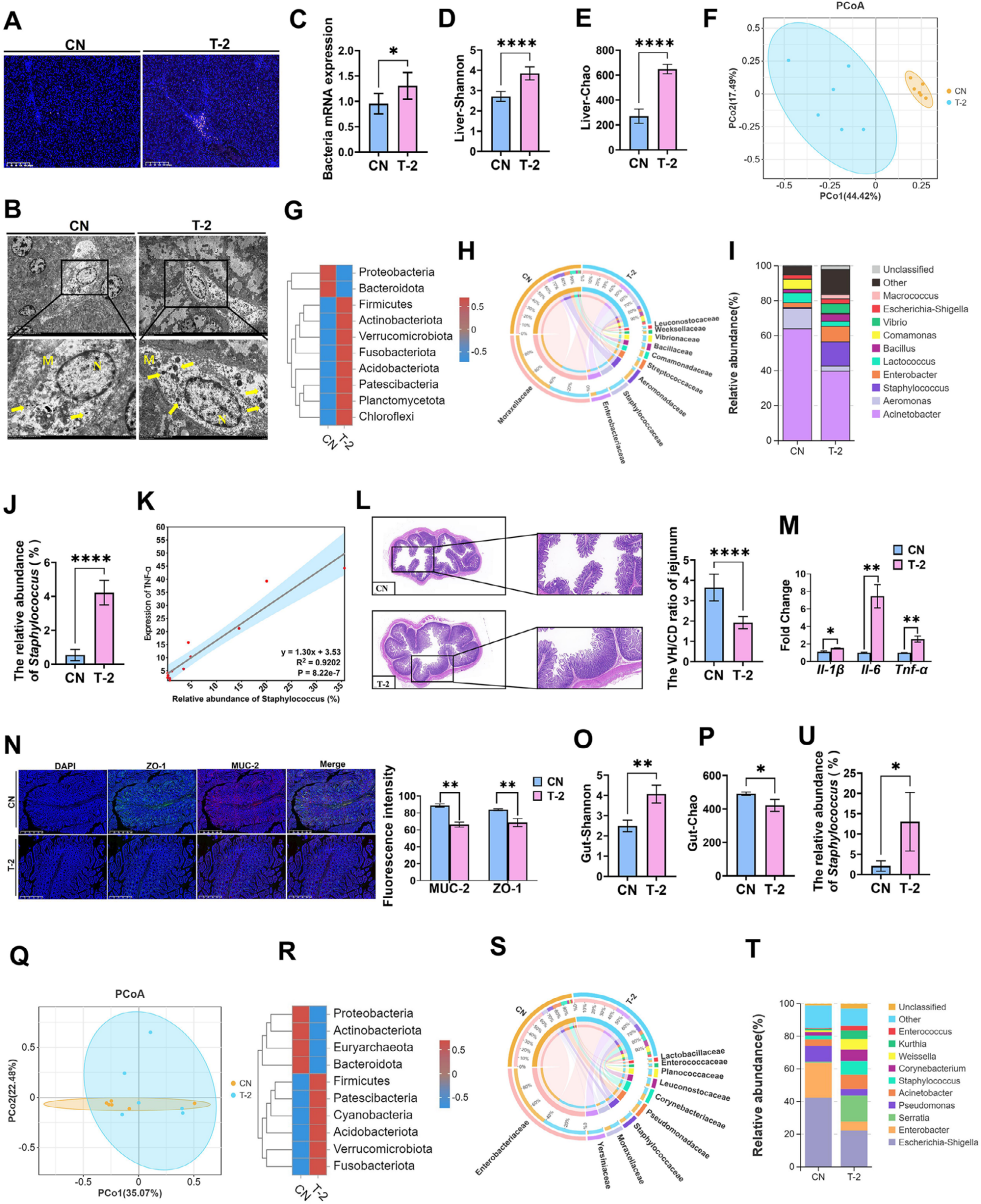
T‐2 toxin‐induced intestinal barrier disruption facilitates gut microbiota translocation. A) Assess the presence of microbes in the liver by 16S FISH. Scale bars, 200 µm. B) Assess the presence of microbes in the liver by TEM. M means endothelial cells, N means Kupper cells, yellow arrow means bacteria. Scale bars, 2 µm. C) Bacterial DNA content of the liver. D,E) Shannon (D) index and Chao (E) index of α diversity of liver microbiota. F) PCoA analysis. G–I) Microbiological composition at the phylum (G), order (H) and genus (I) level of liver microbiota. J) Relative abundance of the genus *Staphylococcus* in the liver. K) Correlation analysis between the abundance of the *Staphylococcus* and Hepatic inflammatory factor TNF‐α. L) H&E staining and VH/CD radio of the jejunum. Scale bars, 500, 200 µm. M) *Il‐1β, Il‐6, Tnf‐α* mRNA levels in the jejunum. N) ZO‐1 and MUC‐2 immunofluorescence staining and intensity analysis. Scale bars, 2000 µm. O,P) The Shannon (O) and Chao (P) index of gut microbiota. Q) PCoA analysis. R–T) Microbiological composition at the phylum (R), order (S), and genus (T) level of gut microbiota. U) Relative abundance of the genus *Staphylococcus* in the gut. VH/CD, villus height/crypt depth. Data represent mean ± SD. *p < 0.05, **p < 0.01, ***p < 0.001, ****p < 0.0001; ns, no significance.

We hypothesized that T‐2 toxin disrupts the intestinal barrier function in piglets, thereby facilitating the translocation of *Staphylococcus* from the gut to the liver. To test this perspective, Further observations using 16S FISH and transmission electron microscopy (TEM) revealed a large number of viable bacteria colonizing the liver in the T‐2 group (**Figure** **2**A,B), a finding that was further validated by PCR (Figure 2C). In addition, the TEM results revealed the presence of autophagosomes and autophagolysosomes, which is consistent with the results shown in Figure 1S, suggesting the occurrence of autophagy (Figure S1N, Supporting Information).

Next, to further characterize the liver microbiome, we conducted 16S rRNA sequencing. The analysis demonstrated significant differences in both α‐diversity and β‐diversity between the two groups (Figure 2D–F). Furthermore, we analyzed the liver microbial composition of both groups at different taxonomic levels (Figure 2G–I). Interestingly, the relative abundance of *Staphylococcus* was significantly higher in the T‐2 group than in the CN group at the genus level (Figure 2J), and a positive correlation was observed between *Staphylococcus* and TNF‐α (Figure 2K).

We conducted a study to investigate the impact of T‐2 toxin on the intestinal barrier of piglets. Piglets exposed to T‐2 toxin exhibited significant jejunal damage, including villus rupture and a reduced villus height‐to‐crypt depth (VH/CD) ratio, as well as increased expression of inflammatory factors (Figure 2L,M). Additionally, the expression of the intestinal barrier proteins ZO‐1 and MUC2 was significantly decreased (Figure 2N). 16S rRNA sequencing of the jejunal contents further revealed a distinct microbial composition between the two groups (Figure 2O–T), with a notable increase in the relative abundance of *Staphylococcus* in the T‐2 group (Figure 2U), consistent with the changes observed in the liver microbiota. These findings suggest that T‐2 toxin may disrupt the intestinal barrier, enabling the translocation of *Staphylococcus* to the liver and thereby influencing liver homeostasis.

### 2.3 T‐2 Toxin Promotes *S. Saprophyticus* Proliferation and Liver Translocation

**Figure 3:**
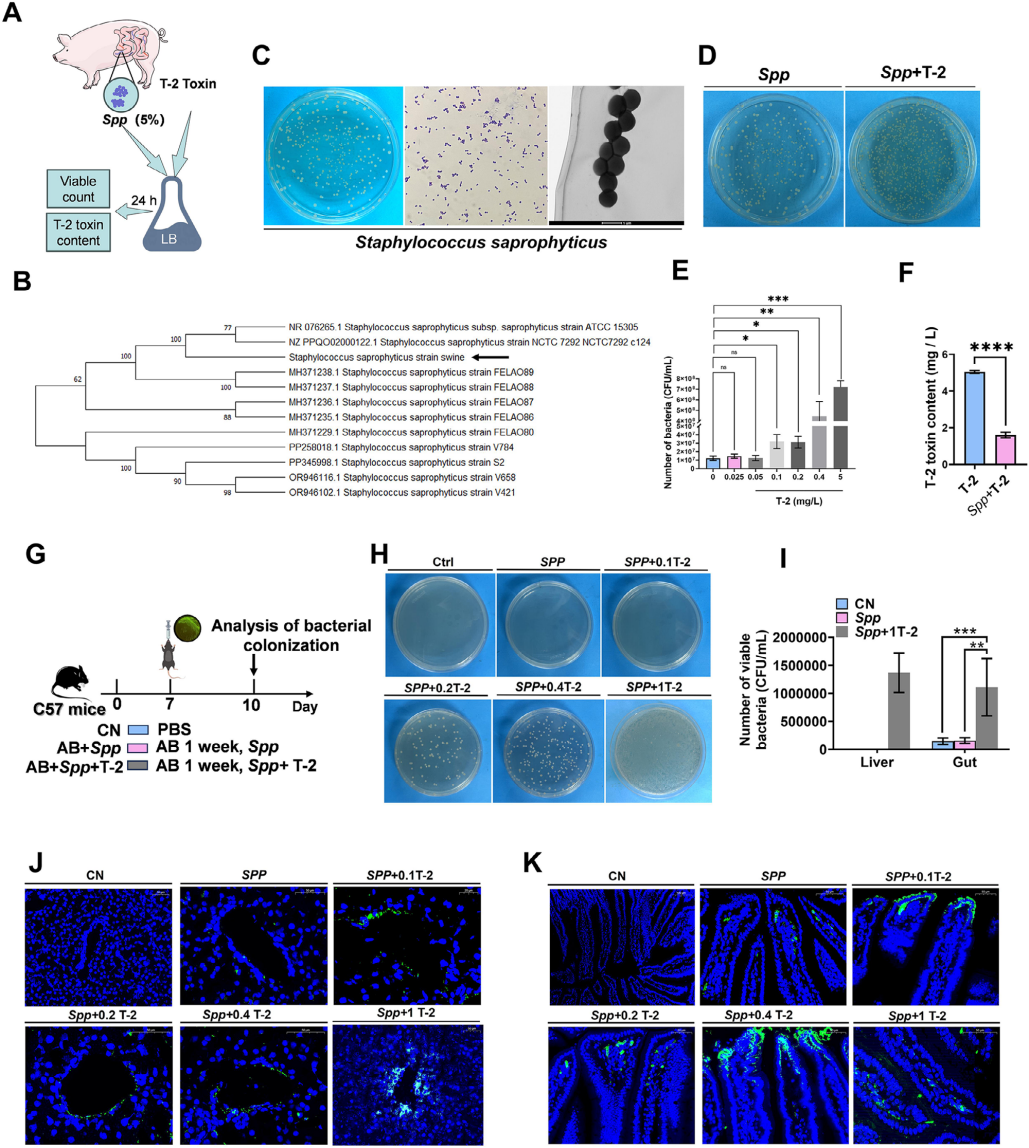
T‐2 toxin promotes *S. saprophyticus* proliferation and liver translocation. A) Experimental scheme of T‐2+*Spp* coculture. B) Evolutional tree of *S. saprophyticus*. C) Morphology, gram stain and electron microscopy images of *S. saprophyticus*. D) Morphology of *S. saprophyticus*. E) Viable count bar charts of *S. saprophyticus* at different T‐2 toxin concentrations. F) Determination of T‐2 toxin in supernatants by HPLC‐MS. G) Experimental scheme of *S. saprophyticus* colonization in antibiotic‐treated mice. H) liver and gut bacterial loads. I) Viable count bar charts of *S. saprophyticus*. J) Immunofluorescence detection of *S. saprophyticus* in the liver of mice. Scale bars, 20 µm. 50 µm. AB, antibiotic cocktail; *Spp*, *S. saprophyticus*. K) Immunofluorescence detection of *S. saprophyticus* in gut of mice. Scale bars, 50 µm. 100 µm. AB, antibiotic cocktail; *Spp*, *S. saprophyticus*. Data represent mean ± SD. *p < 0.05, **p < 0.01, ***p < 0.001, ****p < 0.0001; ns, no significance.

To further confirm whether *Staphylococcus* plays a key role as pathogen following exposure to T‐2 toxin, we isolated and cultured the jejunal contents of piglets, identifying a strain of *Staphylococcus saprophyticus* (*S. saprophyticus*) using 16S rRNA sequencing (**Figure** **3**A,B). The bacterium exhibited a smooth, moist, rounded surface with neat edges. Gram staining confirmed it to be a Gram‐positive organism, and transmission electron microscopy (TEM) revealed spherical cells measuring ≈1 µm in diameter (Figure 3C). To explore the potential interaction between *S. saprophyticus* and T‐2 toxin, we cocultured the identified *S. saprophyticus* with T‐2 toxin in vitro (Figure 3D) and examined bacterial growth and T‐2 toxin content. The results showed that the number of *S. saprophyticus* significantly increased during the coculture with T‐2 toxin at concentrations (0.025–5 mg L^−1^), while the T‐2 toxin concentration significantly decreased (Figure 3E,F). These findings suggest that a sub‐toxic dose of T‐2 toxin could promote the proliferation of *S. saprophyticus*.

Next, we constructed *S. saprophyticus* strains that could stably express green fluorescent protein, and we confirmed their functionality in mice (Figure 3G). Through a series of morphological characterizations and immunofluorescence assays, we found that combining different concentrations of T‐2 toxin (0.025–5 mg L^−1^) and *S. saprophyticus* significantly increased the number of viable bacteria in the liver and intestines (Figure 3H–K). These results preliminarily verify that a sub‐toxic dose of T‐2 toxin can promote the proliferation of *S. saprophyticus* and its translocation to the liver by breaking through the intestinal barrier.

### 2.4 FMT Verified that T‐2‐Derived Gut Microbiota Induced Liver Injury in Piglets

**Figure 4:**
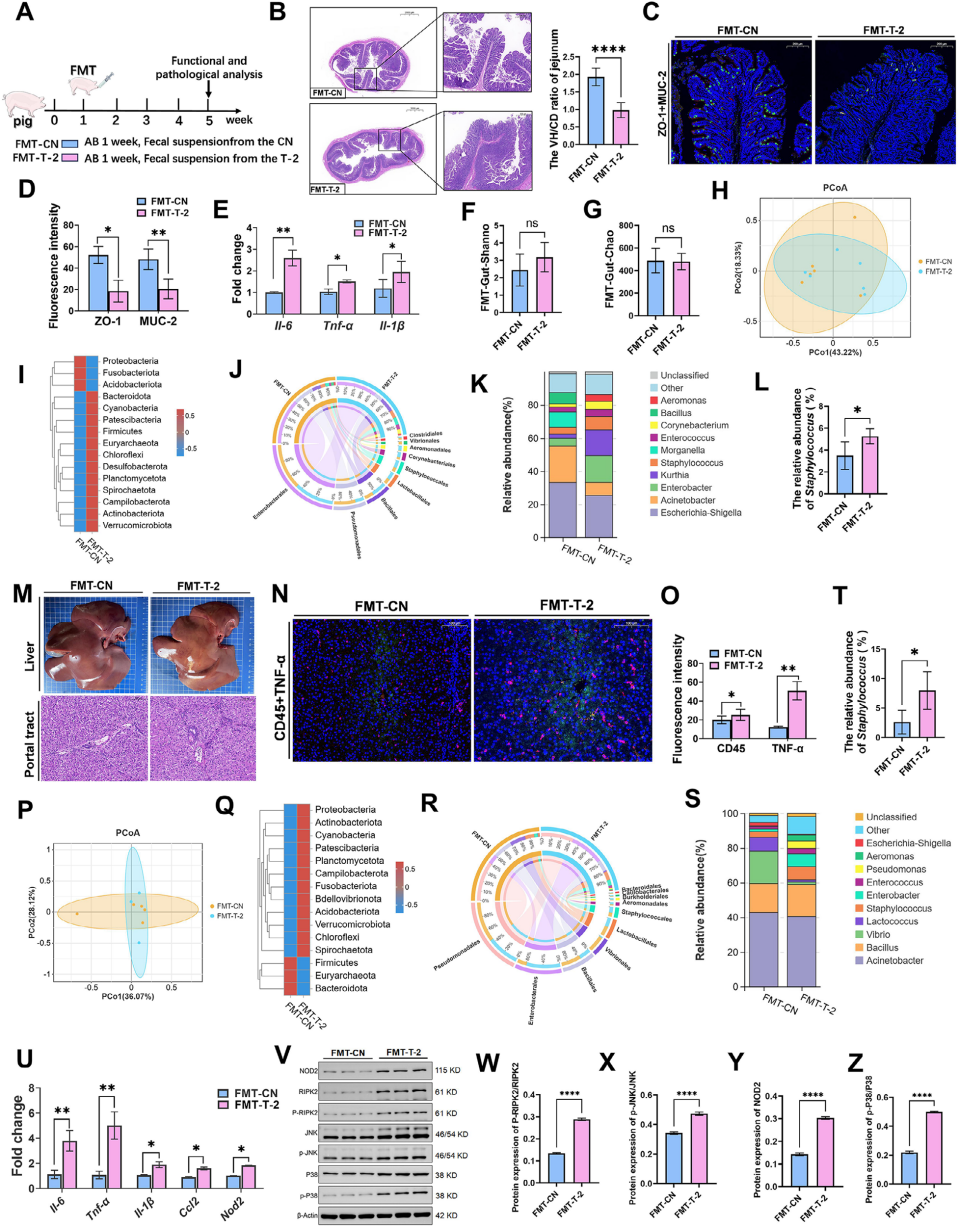
*Staphylococcus* migrates to the liver, aggravating immune damage and inflammation. A) Experimental scheme of FMT in piglets. (*n* = 8 per group). B) H&E staining and VH/CD radio of the jejunum. Scale bars, 2000 µm, 500 µm. C,D) ZO‐1 and MUC‐2 immunofluorescence staining (C) and intensity analysis (D). Scale bars, 500 µm. E) *Il‐6, Tnf‐α, Il‐1β* mRNA levels in the jejunum. F,G) The Shannon (F) and Chao (G) index of the gut. H) PCoA analysis. I–K) Microbiological composition at the phylum (I), order (J), and genus (K) level. L) Relative abundance of *Staphylococcus*. in gut. M) Gross observation and H&E staining of pathological sections of the liver. Scale bars, 100 µm. N,O) CD45 and TNF‐α immunofluorescence staining (N) and intensity analysis (O). Scale bars, 100 µm. P) PCoA analysis. Q–S) Microbiological composition at the phylum (Q), order (R), and genus (S) level. T) Relative abundance of *Staphylococcus* in the liver. U) *Il‐6, Tnf‐α, Il‐1β, Ccl2, Nod2* mRNA levels in the liver. V–Z) Levels of NOD2, RIPK2, JNK, MAPK proteins. AB, antibiotic cocktail; VH/CD, villus height/crypt depth. Data represent mean ± SD. *p < 0.05, **p < 0.01, ***p < 0.001, ****p < 0.0001; ns, no significance.

To further determine the effect of T‐2 toxin derived gut microbiota on immune liver injury in piglets, we performed fecal microbiota transplantation (FMT) (**Figure** **4**A). We found that FMT reproduced the T‐2 toxin phenotype in donor piglets. In particular, the VH/CD ratio in the jejunum of FMT‐T‐2 piglets was reduced (Figure 4B), and ZO‐1 and MUC‐2 expression was also significantly lower than that in the FMT‐CN group (Figure 4C,D). Furthermore, expression levels of inflammatory factors were significantly higher in the FMT‐T‐2 group (Figure 4E). 16S rRNA sequencing analysis revealed no significant differences in α‐ and β‐diversity between the two groups (Figure 4F–H). However, examining the relative abundance of species at different taxonomic levels revealed a notable change in the abundance of the genus *Staphylococcus* (Figure 4I–L). These findings suggest that specific alterations in the microbiota, particularly an increase in *Staphylococcus*, may play a critical role in T‐2 toxin‐induced intestinal barrier disruption and inflammation.

Meanwhile, the FMT‐T‐2 group exhibited significant inflammatory cell infiltration in the portal tract of the liver (Figure 4M), a finding corroborated by immunofluorescence analysis. Furthermore, the expression of CD45 and TNF‐α was markedly increased in the FMT‐T‐2 group compared to the FMT‐CN group (Figure 4N,O). These results suggest that the microbiota from T‐2‐exposed piglets can induce systemic inflammation and immune activation, particularly in the liver. To investigate the microbial differences between the FMT‐T‐2 and FMT‐CN groups, further, 16S rRNA sequencing was performed. The analysis revealed distinct microbial compositions between the two groups, with significant differences observed at various taxonomic levels (Figure 4P–S). Notably, the relative abundance of *Staphylococcus* in the liver and intestine of piglets in FMT‐T‐2 group was significantly higher than that in the FMT‐CN group, which is consistent with the results of previous studies (Figure 4T). Additionally, the results demonstrated that the NOD2 pathway was significantly activated in the FMT‐T‐2 group. This activation was evidenced by the increased expression of NOD2 pathway‐related genes and proteins (Figure 4U–Z). In the previous experiment, we have identified *S. saprophyticus* in the jejunal content of piglets as a T‐2 toxin donor (Figure 3), so we conclude that *S. saprophyticus* can induce immune injury in the liver of piglets.

### 2.5 *S. Saprophyticus* and T‐2 Toxin Synergistically Exacerbate Liver Immune Injury

**Figure 5:**
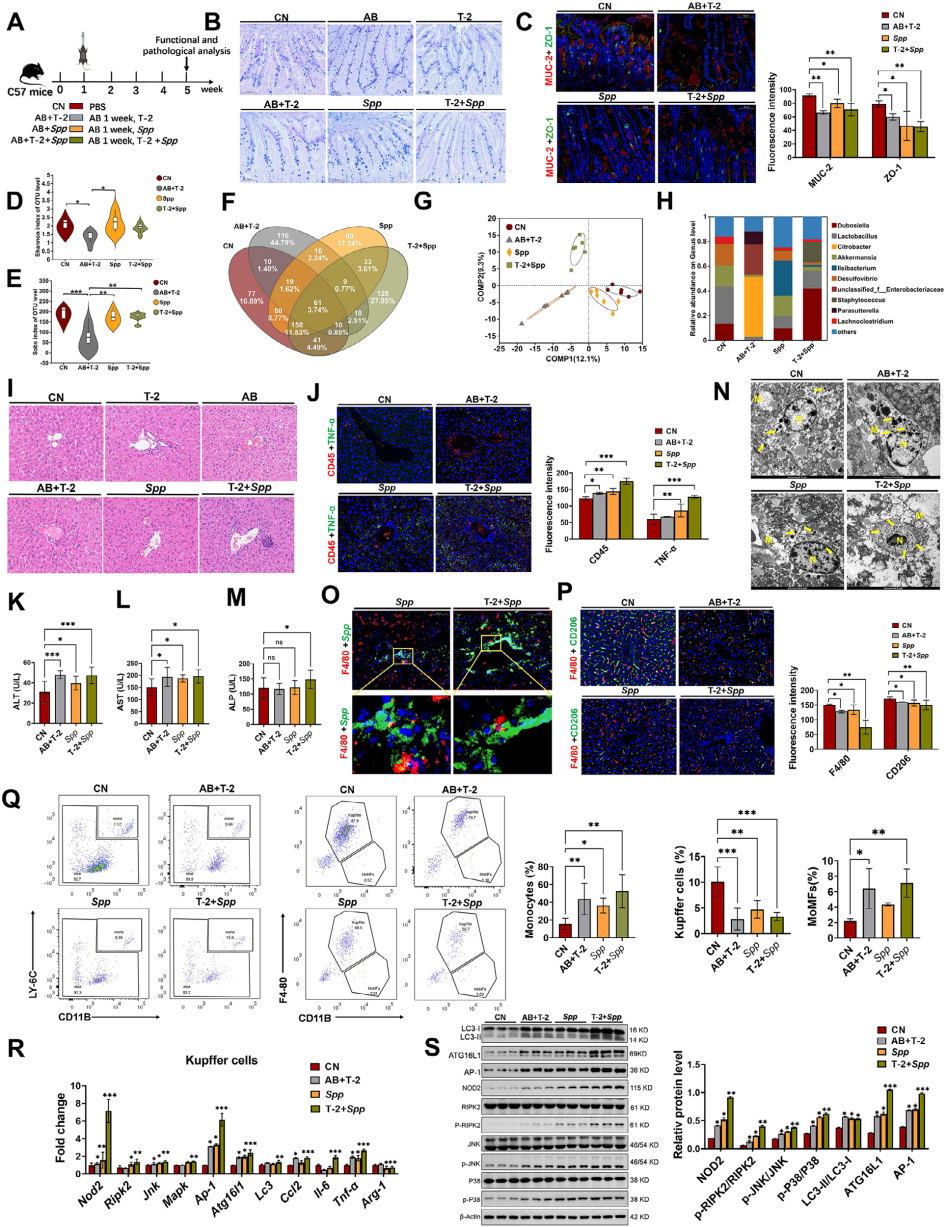
*S. saprophyticus* and T‐2 toxin synergistically increase liver immune function disruption. A) Experimental scheme of bacterial attack test on mice. (*n*=8–10 per group). B) Alisin Blue‐Periodic Acid Schiff (AB‐PAS) staining. Scale bars, 100 µm. C) MUC‐2 and ZO‐1 immunofluorescence staining and intensity analysis. Scale bars, 50 µm. D,E) The Shannon (D) and Sobs (E) index of the gut. F) OUT species Venn diagram. G) PCoA analysis. H) Averaged relative abundance of bacteria at the genus level. I) H&E staining of pathological sections of the liver. Scale bars, 50 µm. J) CD45 and TNF‐α immunofluorescence staining and intensity analysis. Scale bars, 50 µm. K–M) Serum biochemical parameters of ALT (K), AST (L), and ALP (M). N) Bacterial ultrastructure in the perisinusoidal space revealed by TEM. M means endothelial cells, N means Kupper cells, yellow arrow means bacteria. Scale bars, 2 µm. O) Colocalization of *S. saprophyticu*s and F4/80 in liver by immunofluorescence. Scale bars, 100 µm, 20 µm. P) Flow cytometry analysis of monocytes, Kupffer cells and MoMFs. Q) Immunofluorescence double staining of F4/80 and CD206. Scale bars, 50 µm. R) Expression levels of inflammatory factors, the NOD2 pathway, and autophagy‐related genes in Kupffer cells. S) Expression levels of NOD2 pathway and autophagy‐related proteins in hepatic tissue. AB, antibiotic cocktail; *Spp*, *S. saprophyticus*. Data represent mean ± SD. *p < 0.05, **p < 0.01, ***p < 0.001, ****p < 0.0001; ns, no significance.

In view of the above findings that T‐2 toxin could promote the proliferation of *S. saprophyticus* and translocate to the liver by breaking through the intestinal barrier, we constructed models of bacterial infection and toxin exposure, as well as their combined effects, to further analyze the synergistic interactions between *S. saprophyticus* and T‐2 toxin (**Figure** **5**A). The colonization assay revealed that the T‐2+ *Spp* group exhibited significant bacterial aggregation and a marked increase in viable bacterial counts compared to the other three groups (Figure S2A–C, Supporting Information). AB‐PAS staining (Figure 5B) showed that the number of intestinal goblet cells in mice was significantly reduced under the combined effects of *S. saprophyticus* and T‐2 toxin. This observation, coupled with the marked decrease in the expression of intestinal barrier proteins MUC‐2 and ZO‐1, suggested that the intestinal barrier was compromised (Figure 5C; Figure S2D, Supporting Information). Furthermore, 16S rRNA sequencing analysis demonstrated significant differences in the intestinal microbial compositions among the four groups of mice (Figure 5D–G). At the genus level, the relative abundance of *Staphylococcus* was significantly higher in the T‐2+*Spp* group, consistent with previous findings (Figure 5H). H&E staining and immunofluorescence experiments both showed that the synergistic interaction between T‐2 toxin and *S. saprophyticus* intensified the inflammatory response, causing significant immune injury in the liver of the mice characterized by a large numbers of infiltrating inflammatory cells and increased CD45 and TNF‐α expression (Figure 5I,J). Additionally, the levels of AST, ALT, and ALP were significantly elevated in the livers of mice in the T‐2+*Spp* group compared to the CN group (Figure 5K–M). Further TEM and immunofluorescence co‐localization experiments revealed that *S. saprophyticus* aggregation was more pronounced under T‐2 toxin exposure conditions, accompanied by decreased expression of F4/80, a well‐established marker for macrophages, particularly used to identify murine KCs and other tissue‐resident macrophages (Figure 5N,O). These findings suggest that T‐2 toxin not only promotes the proliferation and aggregation of *S. saprophyticus* but also impairs macrophage function, potentially exacerbating bacterial dissemination and liver injury.

To elucidate the mechanism of immune injury caused by the synergistic effect of T‐2 toxin and *S. saprophyticus*, we quantified immune cells using flow cytometry. Compared to the remaining three groups, the T‐2+*Spp* group exhibited an increase in monocytes (CD11B^+^, LY‐6C^+^) and MoMFs (CD11B^+^, F4/80^−^) in the liver. Conversely, the number of KCs (CD11B^−^, F4/80^+^) decreased (Figure 5Q). Immunofluorescence results further confirmed decreased expression of F4/80 and CD206 in the T‐2 + *Spp* group (Figure 5P). We then sorted KCs for qPCR validation. The results showed that the T‐2+*Spp* group exhibited significant upregulation of pro‐inflammatory factors (*Il‐6*, *Tnf‐α*), the chemokine *Ccl2*, NOD2 pathway‐related genes (*Nod2*, *Ripk2*, *Jnk*, *Mapk*, *Ap‐1*), and autophagy‐related genes (*Atg16l1*, *Lc3*) (Figure 5R). High expression of Arg‐1 is an important marker of the alternative activation state in M2 macrophages. However, we found that the expression of Arginase‐1 (*Arg‐1*) was significantly downregulated and that the expression trends of NOD2 pathway‐related proteins (NOD2, RIPK2, JNK, p‐P38, and AP‐1) and autophagy‐related proteins (ATG16L1, LC3) were consistent with those shown in Figure 5R (Figure 5S). To further substantiate the occurrence of autophagy, we conducted TEM analysis and clearly observed the presence of autophagosomes, which are characterized by their double‐membrane vesicles enclosing cytoplasmic material. These observations provide compelling evidence for the activation of autophagic processes (Figure S2E, Supporting Information). Collectively, these results indicate that the synergistic effect of T‐2 toxin and *S. saprophyticus* leads to a decrease in the number of KCs and an increase in the number of monocytes in the liver. This persistent inflammatory response further exacerbates the development and progression of liver disease.

### 2.6 *S. Saprophyticus* Translocates to the Liver, where its Recognition by NOD2

**Figure 6:**
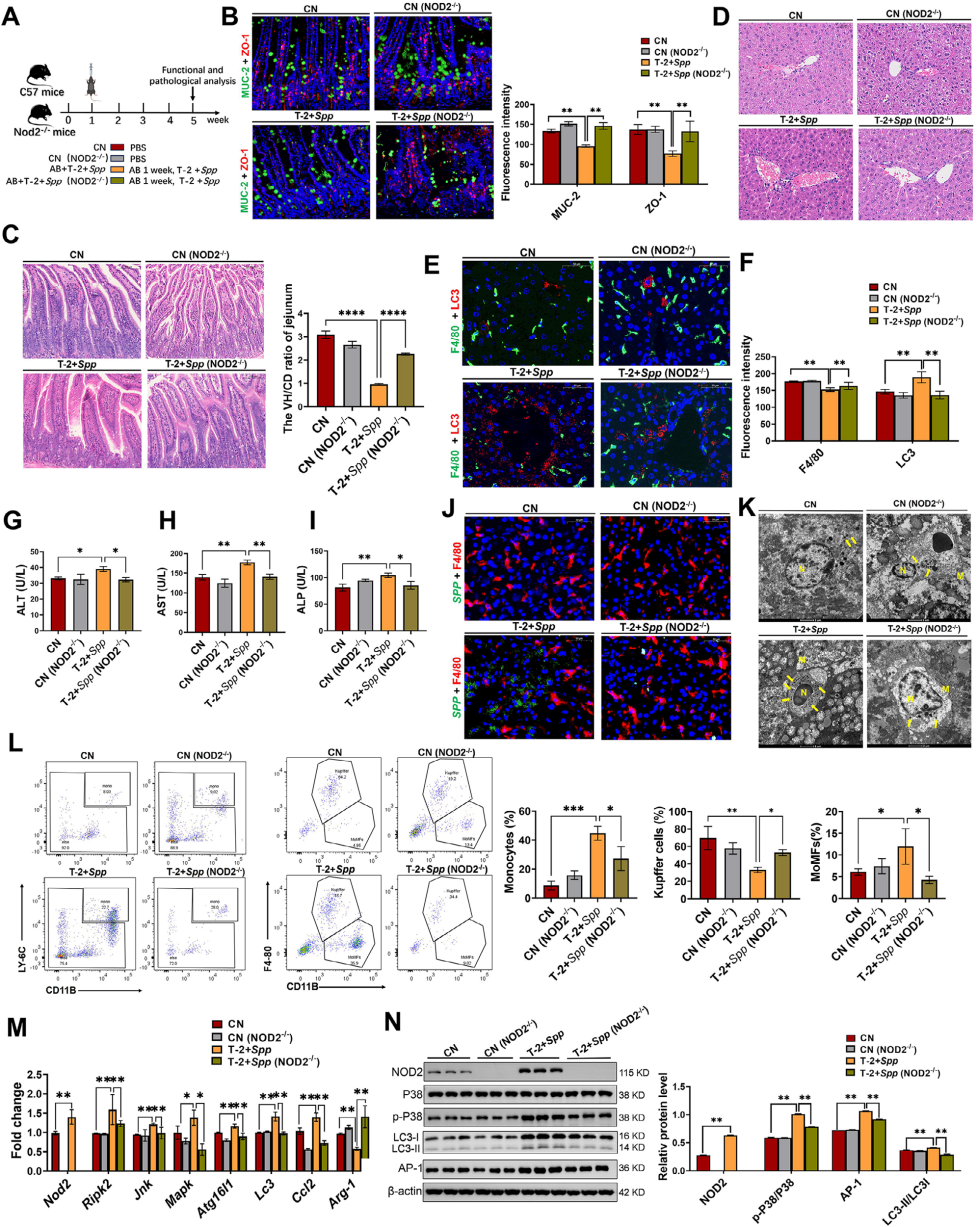
*S. saprophyticus* disrupts the liver immune function, with NOD2 serving as its recognition receptor. A) Experimental scheme of *NOD2^−‐^* mice. (n = 7–8 per group). B) MUC‐2 and ZO‐1 immunofluorescence staining and intensity analysis. Scale bars, 50 µm. C) H&E staining and VH/CD radio of the jejunum. Scale bars, 100 µm. D) H&E staining of pathological sections of the liver. Scale bars, 50 µm. E,F) F4/80 and LC3 immunofluorescence staining (E) and intensity analysis (F). Scale bars, 50 µm. G–I) Serum biochemical parameters of ALT (G), AST (H), and ALP (I). J) Colocalization of *S. saprophyticus* and F4/80 in liver by immunofluorescence. Scale bars, 50 µm. K) Bacterial ultrastructure in the perisinusoidal space revealed by TEM. M means endothelial cells, N means Kupper cells, * means sinusoidal space, yellow arrow means bacteria. Scale bars, 2 µm. L) Flow cytometry analysis of monocytes, Kupffer cells and MoMFs. M) The expression levels of genes associated with the NOD2 pathway and autophagy in hepatic tissue. N) The protein levels related to the NOD2 pathway and autophagy in hepatic tissue. AB, antibiotic cocktail; *Spp*, *S. saprophyticus*. Data represent mean ± SD. *p < 0.05, **p < 0.01, ***p < 0.001, ****p < 0.0001; ns, no significance.

NOD2 is an intracellular pattern recognition receptor that detects bacterial components and plays a key role in defending against bacterial infections and maintaining immune homeostasis.^[^ ^16^ ^]^ Based on the previous results, we hypothesized that *S. saprophyticus* exacerbates T‐2 toxin‐induced hepatic macrophage homeostasis disruption by being recognized by NOD2 during its translocation from the gut to the liver. To test this hypothesis and validate the recognition of *S. saprophyticus* derived immunostimulatory molecules by hepatic NOD2, first, we enzymatically released muramyl dipeptide (MDP) from its cell wall peptidoglycan (PGN) and performed Biotin labeling of MDP. Using Biotin‐labeled MDP, we conducted pull‐down assays with mouse liver lysates. Western blot analysis revealed binding between NOD2 and biotinylated MDP, but not with Biotin alone (Figure S3A, Supporting Information). These results suggest that hepatic NOD2 specifically recognizes *S. saprophyticus* derived MDP, and this interaction serves as the key initiating step triggering downstream signaling pathways. Next, we used NOD2 knockout mice (*NOD2^−/−^*) (**Figure** **6**A). Immunofluorescence analysis revealed that the expression of MUC‐2 and ZO‐1 in the T‐2+*Spp* group was significantly reduced compared to the CN group, indicating impaired integrity of the intestinal barrier in mice (Figure 6B). This finding was further supported by H&E staining, which showed a significant decrease in the VH/CD radio of the jejunum (Figure 6C). Pathological results revealed that the combined effect of T‐2 toxin and *S. saprophyticus* induced extensive inflammatory cell infiltration in the hepatic sinusoids (Figure 6D). As illustrated in Figure 6E–I, the T‐2+*Spp* group increased the fluorescence intensity of the autophagy marker LC3 in liver macrophages, and elevated levels of liver function markers (AST, ALT, and ALP). This confirms that the synergistic effect of T‐2 toxin and *S. saprophyticus* exacerbates liver injury. Notably, all of these alterations were reversed in the T‐2+*Spp* (*NOD2^−/−^*) group. Further immunofluorescence co‐localization of F4/80 and *S. saprophyticus*, along with TEM analysis, showed a significant reduction in the quantity of *S. saprophyticus* in the T‐2+*Spp* (*NOD2^−/−^*) group compared to the T‐2+*Spp* group (Figure 6J,K). Flow cytometry analysis revealed thatthe T‐2+*Spp* group displayed a significant increase in monocytes (CD11B^+^, LY‐6C^+^) and MoMFs (CD11B^+^, F4/80^−^) compared to the CN group, while showing a marked decrease in KCs (CD11B^−^, F4/80^+^) (Figure 6L). Meanwhile, the expression levels of NOD2 pathway‐related genes (*Nod2*, *Ripk2*, *Jnk*, *Mapk*), *Ccl2*, and autophagy‐related genes (*Atg16l1*, *Lc3*) were significantly upregulated, while *Arg‐1* expression was significantly decreased (Figure 6M). The expression trends of NOD2 pathway‐related proteins (NOD2, p‐P38, AP‐1) and autophagy‐related proteins were consistent with the results shown in Figure 6M (Figure 6N). However, these changes were not observed in the T‐2+*Spp* (*NOD2^−/−^*) group. Additionally, the presence of autophagosomes was observed using the TEM (Figure S3B, Supporting Information). In conclusion, our study demonstrates that *S. saprophyticus* translocates to the liver and is recognized by NOD2, inducing a more severe hepatic inflammatory response and immune injury.

### 2.7 XOS Alleviates Liver Immune Function Injury Synergistically Induced by *S. Saprophyticus* and T‐2 Toxin

**Figure 7:**
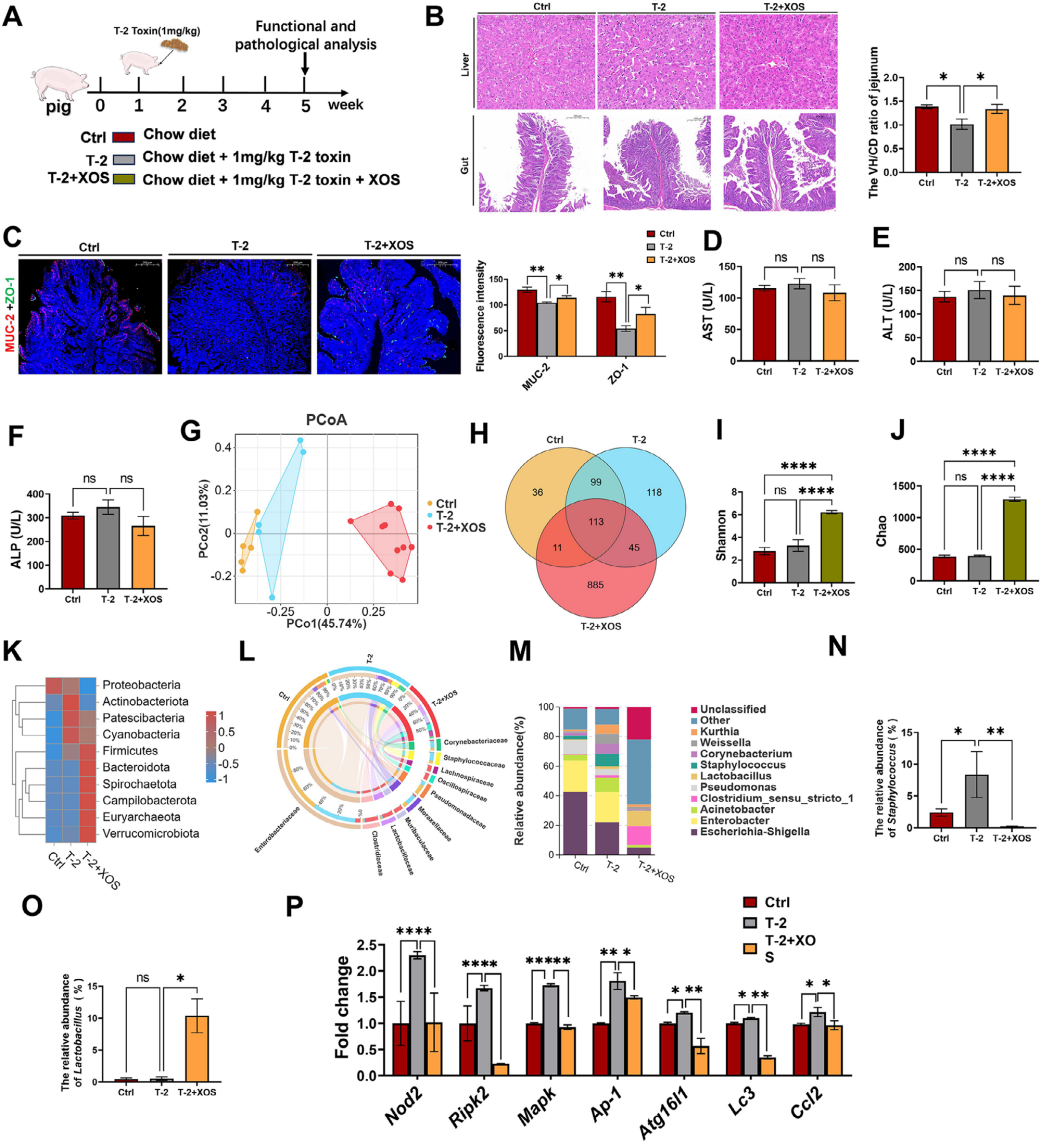
XOS‐mediated protection in piglets. A) Schematic diagram of the experimental design for XOS‐mediated protection in piglets. B) HE staining of the liver and jejunum and the VH/CD ratio of the jejunum. Scale bars, 100, 500 µm. C) ZO‐1 and MUC‐2 immunofluorescence staining and intensity analysis. Scale bars, 500 µm. D–F) Serum biochemical parameters of AST (D), ALT (E), and ALP (F). G) PCoA analysis. H) OUT species Venn diagram. I,J) The Shannon (I) and Sobs (J) index for assessing alpha diversity. K–M) Averaged relative abundance of bacteria at the phylum (K), order (L), and genus (M) level. N) Relative abundance of *Staphylococcus* in the gut. O) Relative abundance of *Lactobacillus* in the gut. P) NOD2 pathway and autophagy‐related genes expression levels. Data represent mean ± SD. *p < 0.05, **p < 0.01, ***p < 0.001, ****p < 0.0001; ns, no significance.

Studies have demonstrated that xylo‐oligosaccharide (XOS), as a high‐quality prebiotic, can enhance host immunity by modulating the gut microbiota.^[^ ^17^ ^]^ To investigate whether XOS exerts a protective effect against liver injury induced by T‐2 toxin exposure, specifically by mitigating *S. saprophyticus* proliferation in the intestine and its subsequent migration to the liver, we established a piglet model (**Figure** **7**A). The results showed that dietary supplementation with XOS alleviated both intestinal and liver damage caused by T‐2 toxin exposure (Figure 7B–F). Furthermore, significant alterations in gut microbial composition were observed (Figure 7G–M). Consistent with previous findings, exposure to T‐2 toxin led to a marked increase in the abundance of *Staphylococcus*, however, this effect was significantly attenuated in the T‐2+XOS group (Figure 7N). Meanwhile, we observed that the abundance of *Lactobacillus* was significantly increased by nearly tenfold in the T‐2+XOS group (Figure 7O). Additionally, the upregulation of the NOD2 pathway and autophagy‐related gene expression induced by T‐2 toxin was notably reversed following XOS intervention (Figure 7P).

**Figure 8:**
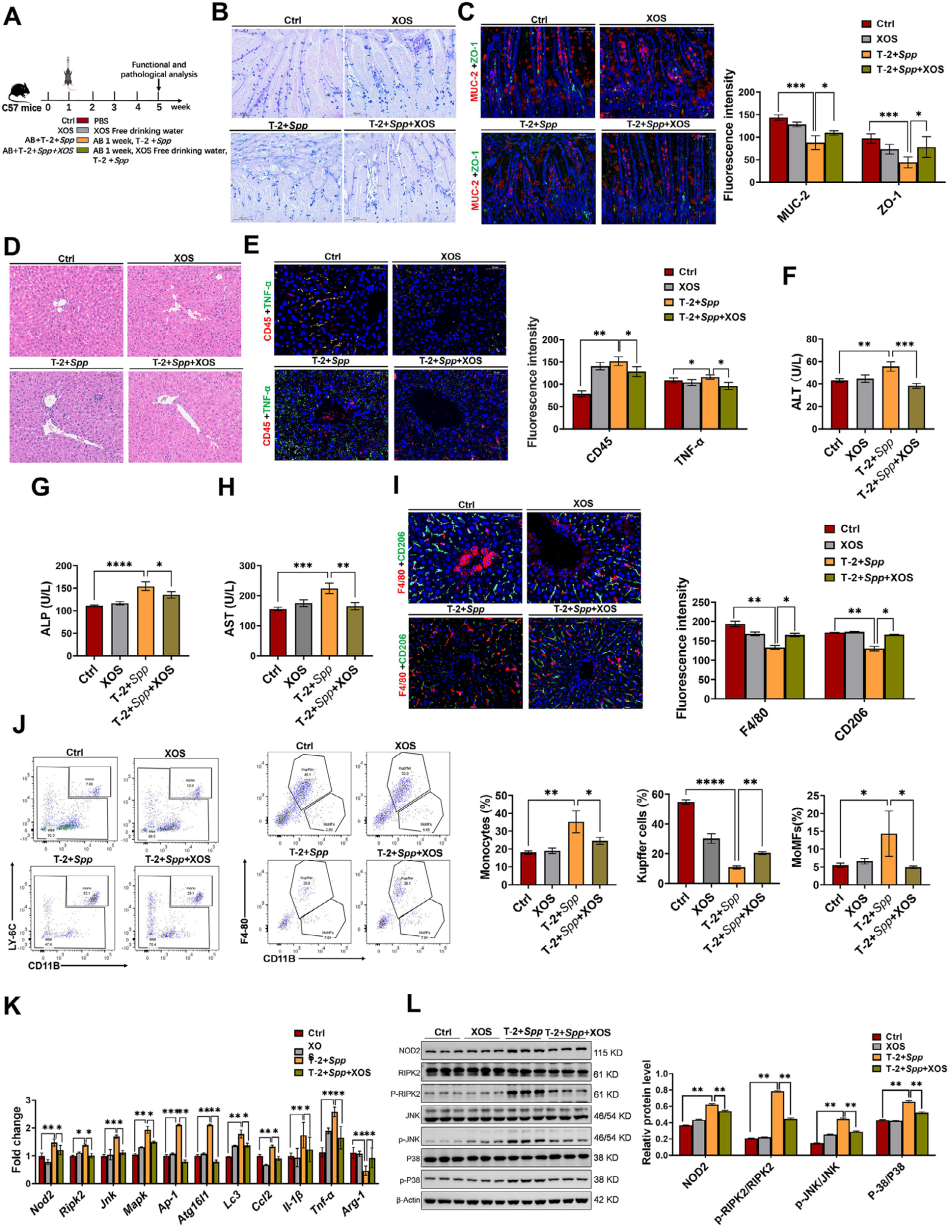
XOS mitigates the liver immune function disruption caused by *S. saprophyticus* and T‐2. A) Experimental scheme of the protective effect of XOS. B) AB‐PAS staining. Scale bars, 100 µm. C) MUC‐2 and ZO‐1 immunofluorescence staining and intensity analysis. Scale bars, 50 µm. D) H&E staining of pathological sections of the liver. Scale bars, 100 µm. E) CD45 and TNF‐α immunofluorescence staining and intensity analysis. Scale bars, 50 µm. F–H) Serum biochemical parameters of ALT (F), ALP (G), and AST (H). I) F4/80 and CD206 immunofluorescence staining and intensity analysis. Scale bars, 50 µm. J) Flow cytometry analysis of monocytes, Kupffer cells and MoMFs. K) The expression levels of inflammatory mediators, NOD2 pathway‐associated genes, and autophagy‐related genes in hepatic tissue. L) The levels of proteins associated with the NOD2 pathway in hepatic tissue. AB, antibiotic cocktail; *Spp*, *S. saprophyticus*. Data represent mean ± SD. *p < 0.05, **p < 0.01, ***p < 0.001, ****p < 0.0001; ns, no significance.

To further validate these findings, a mouse model was developed for subsequent studies (**Figure** **8**A). In this model, we observed that the combination of T‐2 toxin and *S. saprophyticus* induced a phenotype consistent with previous findings. Notably, XOS intervention effectively alleviated the intestinal and liver damage caused by coexposure to T‐2 toxin and *S. saprophyticus*. This was supported by the following key observations: an increase in the number of intestinal goblet cells, a marked decrease in inflammatory cell infiltration (Figure 8B,D), upregulation of MUC‐2 and ZO‐1 expression (Figure 8C), downregulation of CD45 and TNF‐α expression in the liver (Figure 8E), and a significant reduction in liver injury biomarkers (Figure 8F–H). Furthermore, XOS intervention restored the decreased expression of F4/80 and CD206 induced by T‐2 toxin and *S. saprophyticus* coexposure (Figure 8I). Importantly, the alterations in immune cell populations caused by T‐2 toxin and *S. saprophyticus*, such as the increased monocytes (CD11B^+^, LY‐6C^+^) and MoMFs (CD11B^+^, F4/80^−^), as well as decreased KCs (CD11B^−^, F4/80^+^), were significantly reversed following XOS treatment (Figure 8J). Similarly, XOS administration markedly suppressed the activation of the NOD2 pathway and autophagy, thereby alleviating inflammatory responses (Figure 8K,L). In summary, XOS alleviates T‐2 toxin‐ and *S. saprophyticus*‐induced liver injury and inflammation in mice by suppressing M1 macrophage polarization.

## 3 Discussion

*S. saprophyticus* is a prevalent pathogen associated with uncomplicated urinary tract infections in young women and frequently colonizes the lower gastrointestinal tract as a commensal organism. In addition, *S. saprophyticus* can also cause bacteremia and infective endocarditis, but there are few reports.^[^ ^18^ ^]^ To date, the key drivers underlying *S. saprophyticus* proliferation and pathogenicity in animal hosts remain poorly characterized. Disruption of the hepatic macrophage homeostasis compromises host defenses through multiple pathways: impaired pathogen clearance (macrophage dysfunction and bacteremia),^[^ ^19^ ^]^ chronic inflammation with tissue damage (inflammatory cytokine storms and fibrosis), and diminished nonspecific immunity.^[^ ^20^, ^21^ ^]^ In this study, we demonstrated that T‐2 toxin could disrupt the gut barrier and gut microbiota, leading to the escape of *S. saprophyticus* from the intestinal firewall to the liver, and the combined interaction of T‐2 toxin and *S. saprophyticus* to disrupt the hepatic macrophage homeostasis, leading to liver immune injury.

We first demonstrated that T‐2 toxin causes immune damage to the liver. In the piglet model, liver‐function measures showed no significant changes in AST, ALT, or ALP, indicating that the damage was mainly to nonparenchymal liver cells (Figure 1D–F). Liver CD45^+^ nonparenchymal cells were further sorted by flow cytometry and analyzed by single‐cell RNA sequencing. T‐2 toxin significantly reduced the proportion of liver‐resident KCs and significantly increased the proportion of monocytes, but the proportion of other immune cells did not change significantly (Figure 1K,L). KCs are tissue‐resident macrophages in the liver that recognize and clear PAMPs and DAMPs from the portal vein, as well as senescent apoptotic cells. They also maintain liver immune tolerance.^[^ ^22^ ^]^ KEGG enrichment analysis of the gene that were up‐regulated in the T‐2 group revealed significant associations between KCs and monocytes and *Staphylococcus* infection and NOD‐like signaling pathways. We demonstrated that NOD2 pathway was activated through gene and protein validation (Figure 1S,T). Next, we used CellChat to analyze the communication between different cells in the liver. We found that KCs have strong interactions with T cells, monocytes, and B cells. Monocytes, in particular, showed a two‐way signaling with KCs. They also interacted closely with T cells and B cells through autocrine and paracrine signaling (Figure S1K,L, Supporting Information). These findings suggest that monocytes can act as both effector cells and coordinators in inflammation and immune balance. We also discovered that the interaction between KCs and monocytes relies heavily on the CCL chemokine pathway (Figure S1M, Supporting Information). This pathway likely helps recruit monocytes to the liver and controls their differentiation, shaping the local immune response.^[^ ^23^ ^]^ These findings demonstrate that T‐2 toxin induces phenotypic remodeling of hepatic macrophage, characterized by a marked reduction in resident KCs and a concurrent surge in infiltrating monocytes. The accumulated monocytes further differentiate into mature MoMFs, triggering excessive antimicrobial immune responses that exacerbate hepatic inflammatory progression. This immunopathological cascade exhibits correlation with *Staphylococcus* infection severity.

We subsequently characterized hepatic microbial translocation using 16S FISH and TEM. These analyses revealed substantial bacterial colonization in the livers of T‐2 toxin‐exposed piglets. 16S rRNA sequencing confirmed significant proliferation of the *Staphylococcus* genus in both the intestinal and hepatic compartments (Figure 2). This finding was further validated through piglet FMT assays (Figure 4). While prior studies have established that mycotoxins compromise intestinal barrier integrity and gut microbiota homeostasis,^[^ ^14^, ^24^, ^25^ ^]^ our results extend this paradigm by demonstrating T‐2 toxin‐induced structural disintegration of intestinal epithelia and tight junction disruption in both piglets and mouse models. Crucially, we provide mechanistic evidence that T‐2 toxin facilitates *Staphylococcus* translocation along the gut‐liver axis via barrier dysfunction, leading to hepatic colonization that exacerbates dysregulation of the immune microenvironment and perpetuates inflammatory injury.

The link between microbial translocation and an imbalance in hepatic macrophage homeostasis needs to be further explored. Notably, we isolated a strain *S. saprophyticus* and constructed a strain that stably expresses sfGFP, and tested it in a mouse attack experiment. We found that it was able to break through the intestinal barrier, proliferate, and translocate to the liver. Meanwhile, we discovered that *S. saprophyticus* can proliferate in the presence of the different dose of T‐2 toxin (0.025–5 mg L^−1^). This suggests that T‐2 toxin may directly activate the proliferation pathways of *S. saprophyticus* by mimicking growth factor signaling or serving as a metabolic substrate (Figure 3). These findings suggest that *S. saprophyticus* is a key driver of T‐2 toxin‐induced hepatic immune dysfunction.

We suggest that the translocation of S. saprophyticus is closely linked to the dysregulation of hepatic macrophage homeostasis induced by T‐2 toxin, resulting in persistent inflammatory damage. Research by Duan et al. shows that chronic alcohol consumption promotes the growth of *E. faecalis* in the gut, enabling it to breach the intestinal barrier and migrate to the liver. Alcohol also alters the liver macrophage composition and phenotype, while downregulating the expression of complement receptor of the immunoglobulin superfamily (CRIg) on KCs. This reduces the clearance of translocated *E. faecalis*, worsening liver disease.^[^ ^26^ ^]^ Additionally, studies indicate that KCs undergo necroptosis after severe *Listeria* infection, while MoMFs enhance antibacterial immunity through IFN‐γ‐mediated responses.^[^ ^27^ ^]^ Using a wild‐type mouse model (Figure 5), we investigated how *S. saprophyticus* and T‐2 toxin dynamically disrupt hepatic macrophage homeostasis and contribute to liver disease progression. In this model, T‐2 toxin caused damage to liver parenchymal cells, as indicated by a significant rise in liver function markers. Notably, in mice treated with both antibiotics and T‐2 toxin, liver injury‐related enzymes (ALT, AST, ALP) showed significant elevation—a finding inconsistent with the results observed in piglets. This discrepancy may be attributed to different exposure methods: piglets received the toxin through dietary intake, whereas mice were administered the toxin via oral gavage. Furthermore, antibiotic treatment may induce hepatocyte injury, while disruption of the gut microbiota could additionally elevate hepatic T‐2 toxin levels in experimental mice. This may explain why significant elevations in hepatic injury markers were observed in mice, but not in piglets, following T‐2 toxin exposure. Consistent with scRNA‐seq findings in piglets, flow cytometry in the mouse model showed a significant decrease in KC numbers in the livers of mice exposed to both *S. saprophyticu*s and T‐2 toxin (the T‐2+*Spp* group). This drop in KCs numbers is a typical liver response to intracellular bacterial invasion.^[^ ^28^ ^]^ As KCs play a key role in immune surveillance of the liver, a reduction in their numbers likely weakens the liver's ability to clear pathogens and T‐2 toxins. In this study, the reduction of KCs was found to be related to autophagy. At the same time, this reduction allowed peripheral MoMFs to infiltrate and polarize toward the pro‐inflammatory M1 phenotype. These changes were associated with the activation of the NOD2/RIPK2 signaling axis and its downstream pathways. Additionally, the secretion of the chemokine CCL2 further amplified the inflammatory response. These findings indicate that *S. saprophyticus* may synergistically interact with T‐2 toxin to compromise the liver's innate immune barrier by activating the NOD2/RIPK2 axis in KCs, thereby promoting autophagy and chemokine secretion in KCs, inducing aberrant monocyte recruitment and M1 polarization, and ultimately triggering persistent inflammation and immune damage. KCs and MoMFs both play significant roles in the progression and resolution of tissue inflammation and injury across various liver diseases. However, the study of their distinct contributions is complicated by the overlapping phenotypic markers, inflammatory products and inflammatory response mechanisms.^[^ ^29^ ^]^

NOD2 serves as a critical regulator in the pathogenesis of intestinal inflammation.^[^ ^30^ ^]^ While NOD2 deficiency does not directly suppress bacterial translocation, it mitigates intestinal inflammation and barrier impairment, consequently reducing the transfer of gut‐derived bacteria to the liver. To better understand the effects of NOD2 signaling pathway on microbial translocation and liver immune microenvironment imbalance, we created a NOD2 knockout mouse model. After giving wild‐type mice a mix of *S. saprophyticu*s and T‐2 toxin, they showed worse liver injury, fewer KCs, and more MoMFs compared to *NOD2^−/‐^* mice. The NOD2/RIPK2 signaling pathway was activated, along with the release of the chemokine CCL2 (Figure 6). NOD2 is a receptor that recognizes Gram‐positive bacteria like *S. saprophyticus*, which has a peptidoglycan‐rich cell wall. When liver macrophages engulf *S. saprophyticus*, the bacteria break down, releasing muramyl dipeptide (MDP).^[^ ^31^ ^]^ MDP binds to NOD2,^[^ ^32^ ^]^ triggering a signaling cascade that involves receptor‐interacting protein 2 (RIP2), transforming growth factor‐β‐activated kinase 1 (TAK1), and the IκB kinase (IKK) complex. This leads to NF‐κB activation, which induces the expression of pro‐inflammatory genes like *TNF‐α* and *IL‐6*.^[^ ^33^ ^]^ The pathway also activates kinases like p38 and c‐Jun N‐terminal kinase (JNK), driving inflammation and stress responses through the MAPK pathway.^[^ ^34^ ^]^ Additionally, NOD2 interacts with autophagy‐related proteins, including ATG16L1 and LC3, thereby facilitating the regulation of antibacterial autophagy.^[^ ^47^ ^]^ Our findings corroborated these alterations. Notably, T‐2 toxin may also reduce the nutritional support of hepatocytes to KCs (IL‐34‐dependent survival signal) by inhibiting protein synthesis, while its combined exposure with *S. saprophyticus* results in sustained NOD2 signaling activation, leading to homeostasis imbalance and chronic inflammation in hepatocytes.^[^ ^35^, ^36^ ^]^ NOD2 knockout reduced the recognition of *S. saprophyticus*, thereby inhibiting inflammatory response and autophagy, and slowing liver inflammation and immune injury.

XOS is recognized as a novel prebiotic and has been widely used in various fields, including medicine and healthcare, animal feed, and the food industry. Our study demonstrated that the translocation of *S. saprophyticus* induced by T‐2 toxin significantly contributes to liver injury and the impairment of KCs. However, this adverse effect can be prevented and alleviated by the administration of XOS, thereby protecting to both piglets and mice from liver diseases associated with T‐2 toxin and *S. saprophyticus* (Figures 7 and 8). Interestingly, we found that mice in the T‐2 group pretreated with an antibiotic cocktail exhibited more pronounced intestinal damage compared to the CN group. We hypothesized that this was due to antibiotic induced alterations in the composition of the intestinal microbiota,^[^ ^37^, ^38^ ^]^ particularly the potential elimination of key microbial populations that contribute to T‐2 toxin metabolism. This reduction in metabolic capacity may lead to increased accumulation of T‐2 toxin in the body, further exacerbating intestinal damage. *Lactobacillus* is a probiotic known to regulate intestinal homeostasis, enhance gut barrier function, and improve animal immunity, demonstrated significant population increases in both XOS‐treated piglets and mice in the present study.^[^ ^39^, ^40^ ^]^ Therefore, we postulate that the protective effects of XOS may be attributed to two potential mechanisms: suppression of *S. saprophyticus* proliferation and concurrent promotion of *Lactobacillus* abundance. Based on this, XOS has the potential to serve as a promising prebiotic by modulating the gut microbiota and enhancing the abundance of beneficial bacteria (such as *Lactobacillus*), it alleviates hepatic NOD2 over‐activation, thereby indirectly mitigating the damage caused by T‐2 toxin to the host.^[^ ^41^ ^]^

**Figure 9:**
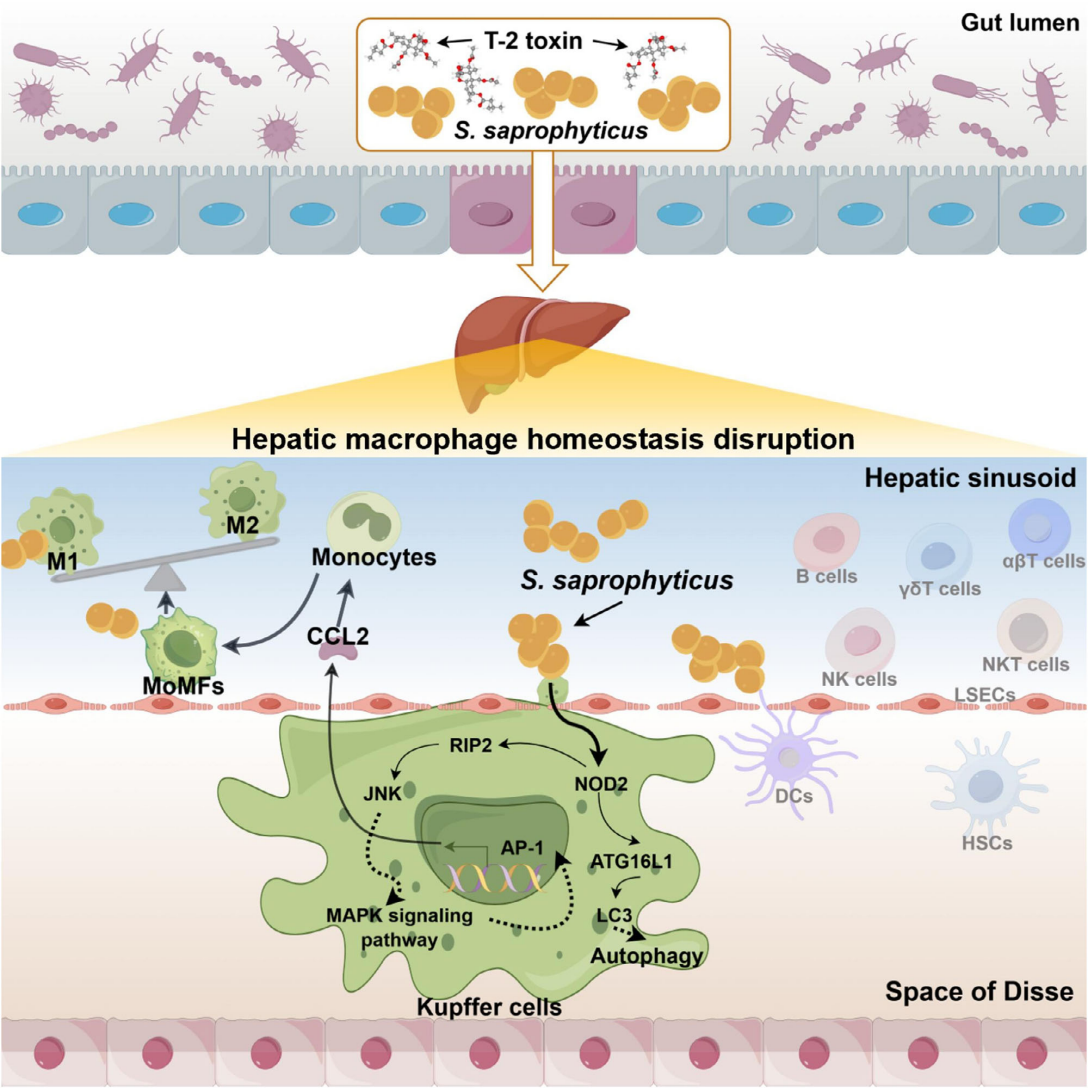
The schematic diagram of T‐2 toxin exploits gut‐derived *Staphylococcus saprophyticus* to disrupt hepatic macrophage homeostasis. The T‐2 toxin facilitates the proliferation of the opportunistic pathogen *S. saprophyticus* in the gut, subsequently enabling its translocation to the liver by breaching the intestinal barrier. The translocated *S. saprophyticus* activates the NOD2 signaling pathway in Kupffer cells (KCs), thereby augmenting autophagic processes within these cells and promoting the secretion of the chemokine CCL2. This cascade of events promotes the recruitment of monocytes and their differentiation into monocyte‐derived macrophages (MoMFs), while also driving the polarization of macrophages towards a pro‐inflammatory M1 phenotype. Collectively, these processes culminate in the disruption of hepatic macrophage homeostasis.

Taken together, our data identify for the first time that T‐2 toxin induces the abnormal proliferation and translocation of *S. saprophyticus* within the intestinal tract, thereby disrupting the homeostasis of hepatic macrophages. Furthermore, it elucidates that the interplay between *S. saprophyticus* and T‐2 toxin as a key driver of the hepatic macrophage homeostasis imbalance (**Figure** **9**). This study highlights the influence of T‐2 toxin on the translocation of *S. saprophyticus* to the liver through the gut‐liver axis and reveals the interaction between opportunistic pathogens, environmental toxins, and hepatic macrophage homeostasis. Furthermore, it proposes potential therapeutic strategies to alleviate mycotoxin‐induced impairment of liver immune function.

## 4 Experimental Section

Key resources for this study are listed in Table S1 in the Supporting Information. Composition of the feed (g kg^−1^) for this study are listed in Table S2 in the Supporting Information. All primers used in this study are listed in Table S3 in the Supporting Information.

### Experimental animals

All animal experiments followed the Animal Welfare and Use Guidelines of China and were approved by the Animal Welfare Committee of Hunan Agricultural University (Approval Number: 2 023 110). Weaned 21‐d‐old Duroc × Landrace × Large triple piglets were bought from Hunan Kangwo Agriculture and Animal Husbandry Co., Ltd (Hunan, China). Wild‐type (WT) C57BL/6J male mice were bought from Hunan SJA Laboratory Animal Co., Ltd (Hunan, China). *NOD2^−/‐^* male mice were bought from Cyagen (Suzhou) Biosciences Inc (Jiangsu, China). All animals were kept under aseptic conditions with free access to food and water. The room temperature was kept at 23±2 °C, and the light/dark cycle was set to 12 h.

### Bacterial Culture

*S. saprophyticus* was isolated from the jejunal contents of piglets. The LB medium was inoculated with 3% of the preserved *S. saprophyticus* glycerol strain, followed by shaking and incubation for 12 h at 37 °C to prepare the bacterial suspension. Viable bacterial counts were determined using the serial dilution method. Subsequently, the concentration of viable bacteria was adjusted to 1 × 10^9^ CFU mL^−1^ using sterile medium. This freshly prepared bacterial suspension was utilized for animal experiments. For the T‐2 and *S. saprophyticus* coculture, *S. saprophyticus* in the logarithmic growth phase were inoculated at 5% into LB liquid medium containing 0–5 mg L^−1^ T‐2 toxin and incubated with shaking at 37 °C for 12–24 h. The T‐2 toxin concentration was quantified using high‐performance liquid chromatography‐mass spectrometry (HPLC‐MS).

### Construction of *S. Saprophyticus* Fluorescent Bacteria

*S. saprophyticus* were activated at 37 °C, inoculated into LB, collected, washed three times with ddH_2_O, twice with 10% glycerol, and finally resuspended in 10% glycerol to prepare competent cells. 90 µL of competent cells were electrotransformed, 50 ng DNA was added, the cells were placed in an ice bath for 5 min, and transferred to a precooled electrorotor (1 mm) and shocked once at 2.9 kV. 1 mL of LB was immediately added, revived at 37 °C for 3 h, and plated on tetracycline resistant (5 µg mL^−1^) AGAR plates for culture overnight. Single colonies were picked and cultured, and plasmids were extracted and sequenced to obtain sfGFP‐*Staphylococcus saprophytic* strains.

### Attack Experiments in Mice

Sixty 8‐week‐old male C57BL/6J mice were first subjected to a 1 week feeding acclimatization. They were then randomly and equally divided into six groups: CN, AB, T‐2, AB+T‐2, AB+*Spp*, and AB+T‐2+*Spp* (*n* = 10). Prior to the formal test, the four groups (excluding CN and T‐2) consumed antibiotic cocktail daily for seven days to eliminate the effects of natural microbiota. During the formal test, the six groups received the following interventions: Mice in the CN and AB groups were gavaged with 0.2 mL of PBS. Mice in the T‐2 and AB+T‐2 groups were gavaged with T‐2 toxin at a dose of 1 mg kg^−1^ d^−1^. Mice in the AB+*Spp* group were gavaged with a *S. saprophyticus* solution (0.2 mL, 1×10⁹ CFU mL^−1^). Mice in the AB+T‐2+*Spp* group were gavaged with 0.2 mL of GFP‐labeled *S. saprophyticus* and 1 mg kg^−1^ of T‐2 toxin daily. The T‐2 toxin dose is determined based on this previous research findings.^[^ ^42^ ^]^ The experiment lasted for 28 d.

### NOD2^−/−^ Mouse Experiment

Fourteen 8‐week‐old male C57BL/6J mice and fourteen 8‐week‐old male *NOD2* ***^−^^/^^−^*** mice were first acclimated for one week before the experiment started. After that, the mice were randomly divided into four groups: the CN group, the AB+T‐2+*Spp* group, the CN (*NOD2* ***^−^^/^^−^***) group, and the AB+T‐2+*Spp* (*NOD2* ***^−^^/^^−^***) group. Each group had seven mice (*n* = 7). The CN group was given a daily oral gavage of 0.2 mL PBS. The AB+T‐2+*Spp* group was given a daily oral gavage of 1 mg kg^−1^ T‐2 toxin mixed with a GFP‐labeled *S. saprophyticus* bacterial solution (1×10^9^ CFU mL^−1^). Before this, the mice in the AB+T‐2+S*pp* group and the AB+T‐2+*Spp* (*NOD2* ***^−^^/^^−^***) group were consumed antibiotic cocktail daily for seven days to eliminate the effects of natural microbiota. The experiment lasted 28 d.

### Xylo‐Oligosaccharide (XOS) Protection Experiments in Mice

Forty 8‐week‐old male C57BL/6J mice were randomly divided into four groups: Ctrl, XOS, T‐2 + *Spp*, and T‐2+*Spp*+XOS, with 10 mice in each group. The Ctrl group was given sterile drinking water, and the XOS group was given drinking water supplemented with 4% XOS. The other two groups were provided with an antibiotic cocktail in their drinking water for 10 d before the experiment started. During the experiment, the T‐2+*Spp* group received a daily gavage of T‐2 toxin (1 mg kg^−1^, adjusted by body weight) and 0.2 mL of *S. saprophyticus* (1×10^9^ CFU mL^−1^). The T‐2+*Spp*+XOS group received the same treatment, with the addition of 4% XOS in their drinking water. The XOS dosage was determined based on the research of Gao et al.^[^ ^17^ ^]^ The experiment lasted 28 d.

### Attack Experiments in Piglets

Sixteen 21‐d‐old weaned Duroc × Landrace × Large White piglets were divided into two groups, each consisting of an equal number of males and females. The first group served as the control group (CN), while the second group was designated as the T‐2 toxin group (T‐2). Following a one‐week acclimatization period, the CN group was fed a standard diet, whereas the T‐2 group received feed supplemented with T‐2 toxin at a concentration of 1 mg kg^−1^. The T‐2 toxin dose was determined based on the previous research findings.^[^ ^42^ ^]^ The experimental duration lasted for 28 d. Piglet feed ingredients are shown in Table S2 in the Supporting Information.

### FMT Experiment in Piglets

Sixteen weaned piglets were divided into two groups. Each group had an equal number of males and females. Antibiotics (1 g kg^−1^ neomycin sulfate and 1.5 g kg^−1^ amoxicillin) were added to the piglets' diet from the start of the acclimatization phase until the seventh day of the main experiment. On the eighth day, the piglets were switched to a standard diet. Feces from the CN and T‐2 groups were mixed with sterile phosphate‐buffered saline at a ratio of 1:5 (feces to PBS). The mixture was filtered through four layers of sterile gauze and poured into a beaker. The filtered liquid was transferred to a 50 mL centrifuge tube and spun at 4000 rpm for 10 min. The solid part at the bottom was mixed with 40 mL of sterile PBS and divided into eight portions of 5 mL each. These portions were used for FMT. The recipient piglets were divided into the FMT‐CN group and the FMT‐T‐2 group. The experiment lasted 28 d.

### XOS Protection Experiments in Piglets

Twenty‐four 21‐d‐old weaned piglets were acclimatized for one week. After that, they were randomly divided into three groups with equal numbers of males and females in each group: control group (Ctrl), T‐2 toxin‐exposed group (T‐2), and T‐2 toxin‐exposed with XOS group (T‐2+XOS) (*n* = 8). The Ctrl group ate standard food. The T‐2 group ate food with 1 mg kg^−1^ T‐2 toxin added. The T‐2+XOS group ate food with both 1 mg kg^−1^ T‐2 toxin and 1% XOS added. The XOS dosage was determined based on the previous study.^[^ ^39^ ^]^ The experiment lasted 28 d.

### Sample Collection and Processing

Mice were fasted but given free access to water for 8 h before dissection. They were euthanized by cervical dislocation under tribromoethanol anesthesia. Piglets were fasted but given free access to water for 8 h and blood was collected from the antecubital vein. The liver and jejunum were carefully separated and quickly frozen on dry ice. All samples were stored at −80 °C until further processing. For gut microbiome analysis, jejunal contents were collected and frozen before analysis.

### Biochemical Parameters of Blood Samples

Blood samples were collected and allowed to clot at room temperature for 30 min. The clotted blood was then centrifuged at 3000 × *g* for 10 min at 4 °C to separate the serum. The supernatant serum was carefully aspirated and transferred to sterile tubes. Serum biochemical parameters, including ALT, AST, and ALP were conducted in accordance with the operational guidelines provided by the Myriad Automatic Animal Serum Biochemistry Instrument.

### Bacterial Isolation and Identification

A spreader was utilized to inoculate the collected intestinal contents onto an LB agar culture plate for three‐zone delineation. Distinct bacterial colonies were selected for general culture, and the resulting bacterial suspension served as a template for amplifying the bacterial 16S ribosomal gene using the universal primers 27F and 1492R. Following gel electrophoresis, the gel was excised and DNA fragments at ≈1500 bp were recovered. These products were subsequently sent to Shanghai Bio‐industry for sequencing. The obtained sequences were analyzed using the NCBI BLAST website to facilitate sequence comparison and identification of bacterial species.

### Histological Evaluation of the Liver and Intestine

Liver and jejunal tissues fixed in 4% paraformaldehyde for histological evaluation. The fixed tissues were initially dehydrated using ethanol and isopropanol, followed by embedding in paraffin and stained with H&E, which were sequentially performed following standard procedures.^[^ ^44^ ^]^ For AB‐PAS staining, paraffin sections were deparaffinized and rehydrated to water. The sections were then sequentially stained with Alcian Blue for 5 min, periodic acid for 15 min, and Schiff's reagent for 30 min, with rinsing in distilled water between each step. After staining, the sections were counterstained with hematoxylin for 2 min and rinsed until the nuclei appeared blue. Finally, the sections were dehydrated, cleared, and mounted using standard protocols. Quantitative analysis was performed using Image J.

### Immunofluorescence Staining

Tissue samples were fixed in 4% paraformaldehyde at 4 °C for 24 h, dehydrated, cleared, and embedded in paraffin wax. The samples were processed following the specific steps described in the previous study.^[^ ^42^ ^]^ Finally, the tissues were stained with 4′,6‐diamidino‐2‐phenylindole (DAPI) solution. Images of the stained tissues were captured using an orthogonal light microscope (Nikon, Tokyo, Japan). Quantitative analysis was performed using Image J.

### Observation of Microstructures by Transmission Electron Microscopy

Fresh liver tissue samples were immediately cut into 1 mm^3^ cubes and fixed in pre‐cooled 2.5% glutaraldehyde (in 0.1 m PBS, pH 7.4) at 4 °C for 4 hours. The tissues were then post‐fixed in 1% osmium tetroxide. After fixation, the samples were dehydrated through a graded series of ethanol and acetone, followed by infiltration with epoxy resin and polymerization. Ultrathin sections (60–80 nm) were cut using a diamond knife, double‐stained with uranyl acetate and lead citrate, and examined under a TEM to visualize subcellular structures.

### Real‐Time Quantitative PCR (RT‐qPCR)

The mRNA expression levels of *Nod2*, *Ripk2*, *Jnk*, *Mapk*, *Atg16l1*, *Lc3*, *Il‐6*, *Il‐1β*, *Tnf‐α*, *Ccl2*, and *Arg‐1* were quantified using qPCR, following the methodology outlined in the previous study.^[^ ^44^ ^]^ The specific primers utilized for this analysis are listed in Table S3 in the Supporting Information.

### Extraction of MDP from *S. Saprophyticus*

The cultured *S. saprophyticus* was boiled in a 4% SDS/Tris‐HCl solution and subjected to ultrasonic treatment. It was then treated sequentially with DNase/RNase and trypsin to obtain peptidoglycan. Following washing, digestion, reduction and acidification, the MDP‐containing wall peptides were captured, eluted and analyzed by LC–MS/MS against synthetic MDP standards. The specific steps were referenced from the study by Girardin et al.^[^ ^45^ ^]^

### Co‐Immunoprecipitation (Co‐IP)

To prepare 100 mg of mouse liver tissue, the tissue was homogenized under liquid nitrogen and lysed with modified RIPA lysis buffer on ice for 30 min. The lysate was centrifuged, and the supernatant was adjusted to a protein concentration of 4–5 µg µL^−1^. A total of 10 µg of Biotin‐MDP was immobilized on streptavidin magnetic beads, which were then incubated with 1 mg of liver lysate at 4 °C for 2 h. According to the protocol, the beads were washed using a magnetic separator, and the washing steps were performed with 50 × 10^−3^ m Tris‐HCl (pH 7.0), 150 × 10^−3^ m NaCl, and 0.1% CHAPS. The bound proteins were eluted by heating at 95 °C in SDS buffer. The eluted samples were subjected to Western blot analysis to detect NOD2.

### Western Blotting Analysis

The protein expression levels of NOD2, RIPK2, p‐RIPK2, JNK, P38, p‐P38, LC3I, LC3II, and AP‐1 were assessed via western blotting, as described in the previous study.^[^ ^42^ ^]^ The signal intensities of the immunoblots were determined using Image J.

### 16S rRNA Sequencing and Data Analysis

Following the extraction of genomic DNA from jejunal contents, the conserved V3–V4 region of the 16S rRNA was amplified using specific primers with barcodes. The PCR‐amplified products were subsequently excised, purified, and quantified using a QuantiFluor fluorometer. The purified amplification products were then combined in equimolar concentrations, ligated to sequencing adapters, and used to construct sequencing libraries, which were sequenced on an Illumina PE250 platform. The specific experimental procedures were performed as described in the previous study.^[^ ^46^ ^]^

### Fluorescence In Situ Hybridization

FISH was performed on paraffin‐embedded liver sections to detect bacterial colonization, following the manufacturer's instructions. A series of steps, including hybridization, washing, restaining, and blocking, were carried out using a hybridization solution containing a Cy3‐labeled EUB338 probe (5′‐GCTGCCTCCCGTAGGAGT‐3′), which targets the 16S rRNA gene. Images were captured using a fluorescence microscope with the appropriate fluorescence channel (excitation: 550 nm/emission: 570 nm). The distribution and signal intensity of the target microorganisms were then analyzed.

### Single‐Cell RNA‐seq

CD45^+^ hepatic nonparenchymal cells from piglets were isolated using flow cytometry. Following quality control of the cell suspension and labeling with the 10× Genomics single‐cell platform, sequencing libraries were constructed. Subsequently, single‐cell high‐throughput sequencing was conducted utilizing the PE150 sequencing mode on the Illumina sequencing platform.

### Flow Cytometry Analysis and Sorting

Liver tissue was excised under aseptic conditions. The tissue was subsequently sectioned and digested using a tissue dissociation solution. The resulting cell suspension was filtered through a 70 µm cell sieve. The hepatocytes, forming the precipitate, were discarded following centrifugation at 4 °C, allowing for the collection of the supernatant. This supernatant was then subjected to a second centrifugation, after which the supernatant was discarded and the precipitate was retained. The precipitate was resuspended in DMEM cell culture medium. A 60% Percoll solution was added to the collected cells, followed by centrifugation to isolate the second layer of liver nonparenchymal cells. These cells were resuspended in DMEM cell culture medium to create a single‐cell suspension for subsequent analyses. The single‐cell suspension was treated with an FC receptor blocker for 5 min. Subsequently, 1 µL each of FITC‐conjugated anti‐Mouse CD45, CD11b, F4/80, LY‐6C, CD86, and CD206 antibodies were sequentially added for incubation. The mixture was gently agitated and incubated at 4 °C for 30 min, protected from light. The cells were then washed twice with a staining buffer. A dead cell activator was added for 10 min, followed by incubation with 7‐AAD for 10 min. Flow cytometry was employed for the analysis and sorting of target cells, and FlowJo software was utilized for subsequent data analysis.

### Statistical Analyses

The results are expressed as mean ± standard deviation (SD). Statistical analyses were conducted using SPSS software, version 25.0 (IBM SPSS, Chicago, IL). The normality of the data was assessed with the Shapiro–Wilk test. For data following a normal distribution, one‐way analysis of variance (ANOVA) was employed. In cases where data were not normally distributed, the Kruskal‐Wallis rank‐sum test was utilized. Post hoc analyses were performed using Dunn's multiple comparison test. *P* values for each figure and panel are indicated in the figure legends.

## Conflict of Interest

The authors declare no conflict of interest.

## Author Contributions

Y.Z. and L.X. contributed equally to this work. Conceptualization, Y.Y.Z., and J.W.; methodology and experiments, J.W., Y.Y.Z., L.X., F.R.G., X.Y.L., J.Y.Q., Q.R.X., S.S.Y., J.S., J.P.W., and X.X.H.; data analysis, R.M.R., L.X., and F.R.G.; funding acquisition, J.W. and L.X.W.; writing – original draft, Y.Y.Z., L.X., and F.R.G.; writing – review and editing, J.W., L.X.W., L.X., S.P.L., C.Z., J.W., R.F.L., Z.H.Y., J.E.Y., and Y.L.Y.; resources, Y.L.Y.; project administration, J.W., L.X.W., and Y.L.Y.; supervision, J.W., L.X.W.

## Supporting information



Supporting Information

## Data Availability

The data that support the findings of this study are available from the corresponding author upon reasonable request.
