# Supplementary material for: T‐2 Toxin Exploits Gut‐Derived Staphylococcus Saprophyticus to Disrupt Hepatic Macrophage Homeostasis
Source: Adv Sci (Weinh). 2025 Sep 6;12(43):e12828. doi: 10.1002/advs.202512828 (PMC12631815; doi:10.1002/advs.202512828)
Supplement: Supplementary file 1 — Supporting Information [file ADVS-12-e12828-s001.docx]

Supplementary Materials

**T-2 toxin exploits gut-derived *Staphylococcus saprophyticus* to disrupt hepatic macrophage homeostasis**

*Yuanyuan Zhu* ^1, †^, *Liu Xu* ^1, †^, *Fangrui Guo* ^1^, *Jianyu Qu* ^1^, *Xiangyan Liu* ^1^, *Qiurong Xu* ^1^, *Jie Sheng* ^1^, *Jiangping Wang* ^1, 2^, *Xiaohong Xie* ^1, 2^, *Ruimin Ren* ^3, 4^, *Chuan Zhou* ^1, 4^, *Sisi Yan* ^5^, *Shuiping Liu* ^1^, *Zhihang Yuan* ^1^, *Rongfang Li* ^1^, *Jing Wu* ^1^, *Jine Yi* ^1^, *Yulong Yin* ^4, 6, 7, *^, *Lixin Wen* ^1, 8, *^, *Ji Wang* ^1, *^

1 Hunan Engineering Research Center of Livestock and Poultry Health Care, College of Veterinary Medicine, Hunan Agricultural University, Changsha 410128, China

2 Changsha Lvye Biotechnology Co., Ltd., Changsha 410100, China

3 Key Laboratory of Livestock and Poultry Resources (Pig) Evaluation and Utilization, Ministry of Agriculture and Rural Affairs, College of Animal Science and Technology, Hunan Agricultural University, Changsha 410128, China

4 Yuelushan Laboratory, Changsha 410128, China

5 School of Basic Medicine, School of Public Health, Hengyang Medical School, University of South China, Hengyang 42100l, China

6 Institute of Subtropical Agriculture, Chinese Academy of Sciences, Changsha 410125, China

7 Institute of Yunnan Circular Agriculture Industry, Pu'er 665000, China

8 Hunan Collaborative Innovation Center of Animal Production Safety, Changsha 410128, China

† These authors contributed equally.

*Corresponding author: Yulong Yin (yinyulong@isa.ac.cn), Lixin Wen (sfwlx8015@sina.com), Ji Wang (wangjics@163.com)

**Table 1. List of key resources used in this study.**

| **REAGENT or RESOURCE** | **SOURCE** | **IDENTIFIER** |
| --- | --- | --- |
| β-actin (Rabbit) | Proteintech Group | Cat#81115-1-RR  RRID: AB_2923704 |
| NOD2(Rabbit) | Abclonal | Cat #A25228  415992 |
| RIPK2(Rabbit) | affinity | Cat #DF6967  RRID: AB_2838923 |
| p-RIPK2(Rabbit) | affinity | Cat #AF0049  RRID: AB_2834094 |
| JNK(Rabbit) | Servicebio | Cat #GB114321-100 |
| p-JNK(Rabbit) | Servicebio | Cat #GB15018-100 |
| P38(Rabbit) | Proteintech Group | Cat#14064-1-AP  RRID: AB_2878007 |
| p- P38(Rabbit) | Proteintech Group | Cat#28796-1-AP RRID:AB_2918205 |
| LC3(Rabbit) | abcam | Cat #ab51520  RRID: AB_881429 |
| ATG16L1(Rabbit) | affinity | Cat #DF3825  RRID: AB_2836182 |
| AP-1(Rabbit) | affinity | Cat #AF6090  RRID: AB_2834984 |
| NOD2(Pig) | Abmart | Cat #TD12125 |
| RIPK2(Pig) | Proteintech Group | Cat #15366-1-AP  RRID: AB_2253650 |
| JNK(Pig) | affinity | Cat #AF6318  RRID: AB_2835177 |
| p-JNK(Pig) | affinity | Cat #AF3318  RRID: AB_2834737 |
| P38(Pig) | affinity | Cat #AF6456  RRID: AB_2835277 |
| p- P38(Pig) | affinity | Cat #AF4001  RRID: AB_2835330 |
| HRP labeled goat anti rabbit | Kirkegaard & perry laboratories | Cat #074-1506 |
| APC-Cy7 Rat Anti-Mouse CD45(30-F11) | BD Pharmingen | Cat #557659 |
| FITC Rat anti-Human/Mouse CD11b | Absin | Cat#abs1850015-100T |
| PE Rat Anti-Mouse CD206(Y17-505) | BD Pharmingen | Cat #568273 |
| BV421 Rat Anti-Mouse F4/80(T45-2342) | BD Pharmingen | Cat #565411 |
| APC Rat Anti-Mouse Ly-6C(AL-21) | BD Pharmingen | Cat #560595 |
| PE-Cy7 Rat anti-Mouse CD86 Antibody | Absin | Cat#abs1850145-100T |
| PE-Cy7 Rat Anti-Mouse CD86(GL1) | BD Pharmingen | Cat #560582 |
| T-2 toxin | Macklin | 21259-20-1 |
| LB medium | Oxoid | CM0996B |
| Glycerol | Hushi | 56-81-5 |
| PVDF membrane(0.45μm) | Millipore | IPVHO0010 |
| PVDF membrane(0.22μm) | Millipore | ISEQ00010 |
| Xylo-oligosaccharide  (Extracted from *Miscanthus lutarioriparius*) | Changsha Lvye Biotechnology | N/A |
| Tribromoethanol | MeilunBio | 75-80-9 |
| Aspartate aminotransferase Assay Kit | Mindray | P/N:105-000443-00 |
| Alanine aminotransferase Assay Kit | Mindray | P/N:105-000442-00 |
| Alkaline phosphatase Assay Kit | Mindray | P/N:105-000444-00 |
| BeyoMag™ Streptavidin Magnetic Beads | Beyotime Biotechnology | P2151 |
| Biotin-LC-NHS | Beyotime Biotechnology | P0632M |
| Ultrasensitive ECL Chemiluminescence Kit | Beyotime | PO018M |

**Table S2 Composition of the feed (g / kg).**

| Composition | Content (%) |
| --- | --- |
| Corn | 28.98 |
| Extruded Corn | 15 |
| Broken Rice | 15 |
| Extruded Soybean | 13 |
| Whey Powder | 8 |
| Bean Meal | 4.5 |
| Glucose | 4 |
| Fish Meal | 3 |
| Hydrolyzed Wheat Protein | 3 |
| Soybean Oil | 1.5 |
| Calcium Formate | 0.8 |
| Montmorillonite | 0.5 |
| Calcium Biphosphate | 0.5 |
| Lysine | 0.65 |
| Methionine | 0.16 |
| Threonine | 0.24 |
| Antioxidant | 0.1 |
| Tryptophan | 0.07 |
| Premix^1^ | 1 |
| Total | 100 |
| Nutrition Level Measured Value（%） |  |
| Total Energy(MJ/Kg） | 18.78 |
| Dry Matter | 89.38 |
| Crude Protein | 17.39 |
| Coarse Ash | 3.9 |
| Calcium | 0.69 |
| Total Phosphorus | 0.54 |
| Lysine | 1.23 |
| Threonine | 0.9 |
| Methionine | 0.57 |
| Tryptophan | 0.26 |

^1^Premixes are contained per kilogram of ration: vitamin A 15,000 IU, vitamin B1 4.32 mg, vitamin B2 12 μg, vitamin B6 4.86 mg, vitamin B12 30 mg, vitamin D3 4,500 IU, vitamin E 72.5 IU, vitamin K3 4.5 mg, biotin 0.48 mg, folic acid 1.764 mg, D-pantothenic acid 19.32 mg, nicotinamide 41.58 mg, Cu 110 mg, Fe 165 mg, Mn 60 mg, Zn 80 mg, I 0.8 mg, Se 0.30 mg.

**Table S3 Primers for qPCR**

| Species | Primer | Forward (5'to 3') | Reverse (5' to 3') |
| --- | --- | --- | --- |
| ^Mouse^ | *^β-actin^* | ^CATCCGTAAAGACCTCTATGCCAAC^ | ^ATGGAGCCACCGATCCACA^ |
|  | *^Nod2^* | ^CCTGGTACGTGCCCAAAGTAG^ | ^GCCAAGTAGAAAGCGCAAA^ |
|  | *^NF-κB^* | ^GCATTCTGACCTTGCCTATCT^ | ^CTCCAGTCTCCGAGTGAAGC^ |
|  | *^Tnf-α^* | ^CCCTCACACTCAGATCATCTTCT^ | ^GCTACGACGTGGGCTACAG^ |
|  | *^Il-6^* | ^CTGCAAGAGACTTCCATCCAG^ | ^AGTGGTATAGACAGGTCTGTTGG^ |
|  | *^Jnk^* | ^AGCAGAAGCAAACGTGACAAC^ | ^GCTGCACACACTATTCCTTGAG^ |
|  | *^Ccl2^* | ^TTAAAAACCTGGATCGGAACCAA^ | ^GCATTAGCTTCAGATTTACGGGT^ |
|  | *^Ccr2^* | ^ATCCACGGCATACTATCAACATC^ | ^CAAGGCTCACCATCATCGTAG^ |
|  | *^Ap-1^* | ^CCTTCTACGACGATGCCCTC^ | ^GGTTCAAGGTCATGCTCTGTTT^ |
|  | *^Atg16l1^* | ^AGCAGCTACGAGACGCTCT^ | ^CGCATCGAAGACATACGAGG^ |
|  | *^Lc3^* | ^GACCGCTGTAAGGAGGTGC^ | ^CTTGACCAACTCGCTCATGTTA^ |
|  | *^Mapk^* | ^TGACCCTTATGACCAGTCCTTT^ | ^GTCAGGCTCTTCCACTCATCTAT^ |
|  | *^Arg-1^* | ^CTCCAAGCCAAAGTCCTTAGAG^ | ^AGGAGCTGTCATTAGGGACATC^ |
| ^Pig^ | *^Gapdh^* | ^AGGGCATCCTGGGCTACACT^ | ^TCCACCACCCTGTTGCTGTA^ |
|  | *^Mapk^* | ^AGCTTCAGCAGATTATGCGTC^ | ^ATCTTCGGCATCTGGGTCAA^ |
|  | *^Nod2^* | ^CAGCTGGACCACAACTCTGT^ | ^CTAAGGCTTGAGCACCCACA^ |
|  | *^Ccl2^* | ^CGTGGCTGCAATTTTCCCAT^ | ^TCCATGCCTGACCCTCTTTTG^ |
|  | *^Ccr2^* | ^ACAGGGTGTGTCCGATTCAA^ | ^GTCCCTGCATCTCTAGCACT^ |
|  | *^NF-κB^* | ^ATCGCGTCCTTTCCACAGTT^ | ^ACCTTTGAGATGTGCTGCGA^ |
|  | *^Tnf-α^* | ^CGTGAAGCTGAAAGACAACCAG^ | ^GATGGTGTGAGTGAGGAAAACG^ |
|  | *^Il-10^* | ^CATCCACTTCCCAACCAGCC^ | ^CTCCCCATCACTCTCTGCCTTC^ |
|  | *^Il-6^* | ^CCTCTCCGGACAAAACTGAA^ | ^TCTGCCAGTACCTTGCT^ |
|  | *^Il-1β^* | ^CAGCTGCAAATCTCTCACCA^ | ^TCTTCATCGGCTTCTCCACT^ |
|  | *^Atg16l1^* | ^CAGAGTGCTTTAGAGGCTTTGC^ | ^AGTTTGCCTTCACAGCACCC^ |
|  | *^Lc3^* | ^GACCAGCACCCCAGCAAAAT^ | ^GACCTTGACCTTTCCAGCCAC^ |
| ^Bacteria^ | *^Gapdh^* | ^CCTCCCCGTTCGACAGACA^ | ^GCCAAATCCGTTCACTCCGAC^ |
|  | *^16S rRNA^* | ^ACTCCTACGGGAGGCAGCAG^ | ^GGACTACHVGGGTWTCTAAT^ |


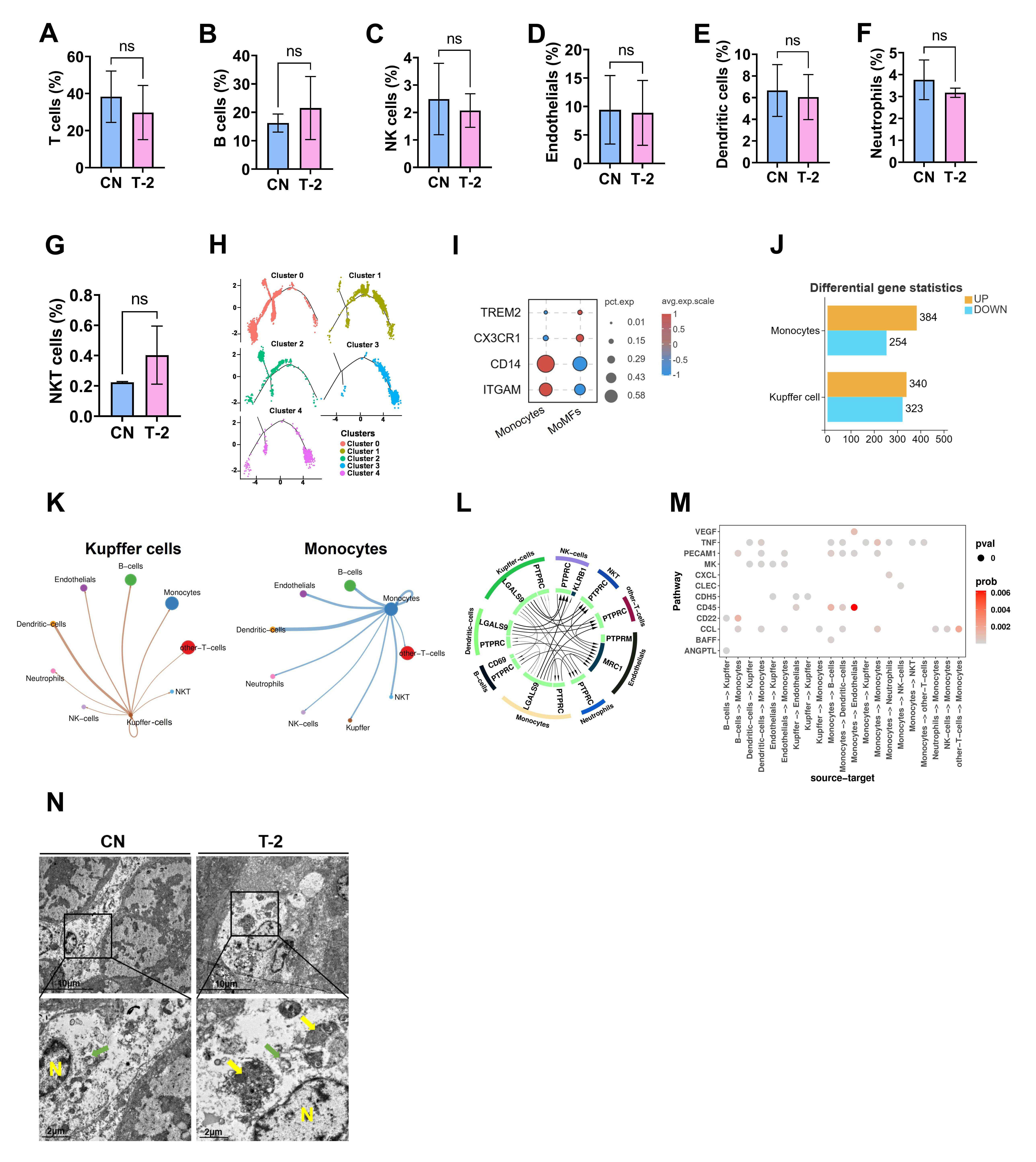


**Figure S1.** T-2 toxin exposure disrupts the liver immune function. **A-G)** Changes in the proportions of cell subpopulations. **H)** Distribution of cell subpopulations along the differentiation trajectory. **I)** Bubble plot of marker genes for re-clustered Monocytes. **J)** Statistical analysis of differentially expressed genes (DEGs) between Monocytes and Kupffer cells. **K)** Comparative analysis of Kupffer cells and Monocytes. **L)** Ligand-receptor interaction network. **M)** Bubble plot of differential probabilities for ligand-receptor pairs. **N)** Ultrastructure in the perisinusoidal space revealed by TEM. N means nucleus, green arrow means autophagosome, yellow arrow means autolysosome. Scale bars, 2 μm. Data represent mean ± SD. **p* < 0.05, ***p* < 0.01, ****p* < 0.001, *****p* < 0.0001; ns, no significance.


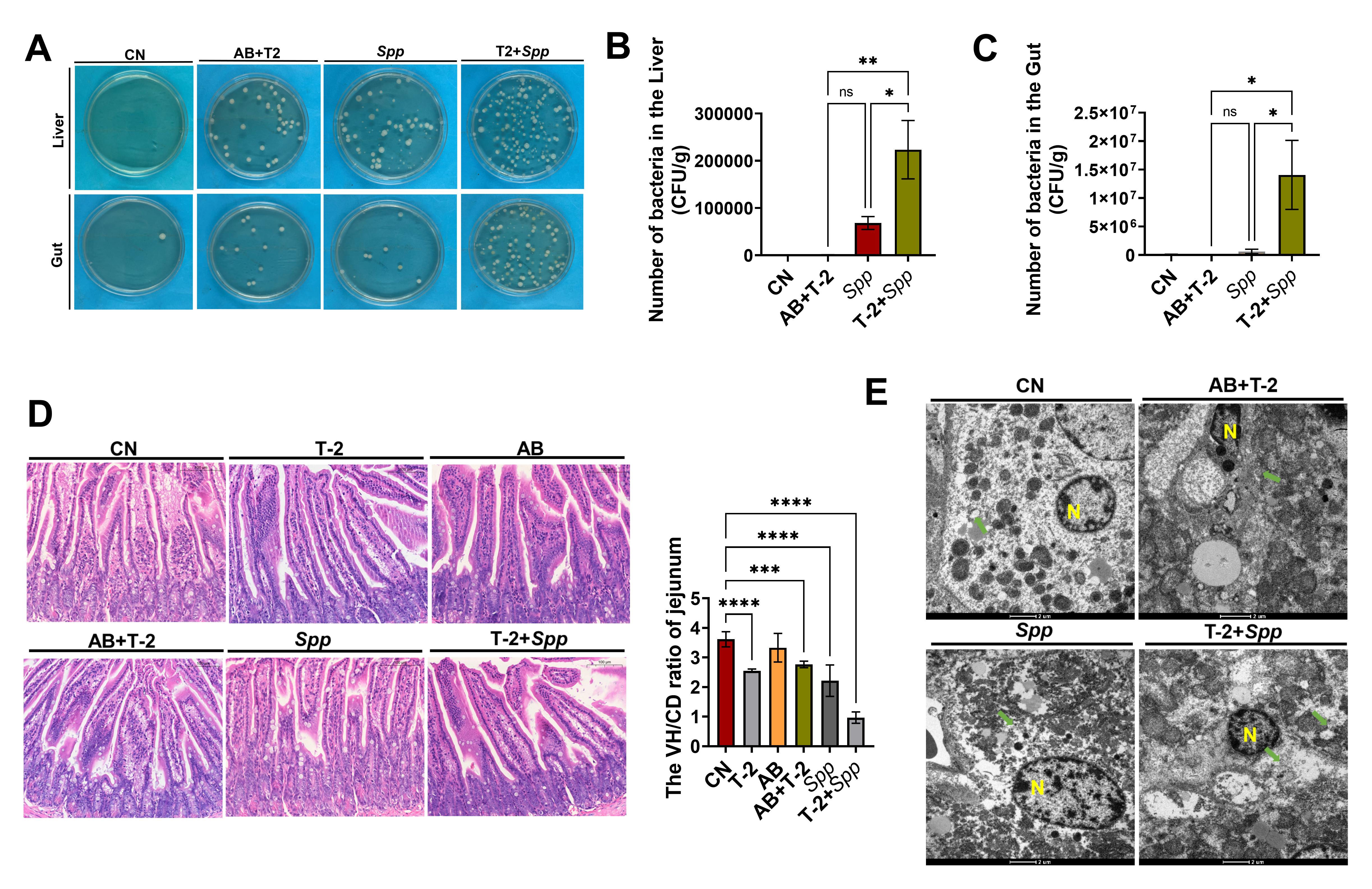


**Figure S2.** The synergistic effect of *S. saprophyticus* and T-2 toxin. **A-C)** Colony counting plate chart of *S. saprophyticus* in the liver and gut of mice. **D)** H&E staining and VH/CD radio of the jejunum. Scale bars, 100μm. **E)** Ultrastructure in the perisinusoidal space revealed by TEM. N means nucleus, green arrow means autophagosome. Scale bars, 2 μm. AB, antibiotic cocktail; *Spp*, *S. saprophyticus*. VH/CD, villus height/crypt depth. Data represent mean ± SD. **p* < 0.05, ***p* < 0.01, ****p* < 0.001, *****p* < 0.0001; ns, no significance.


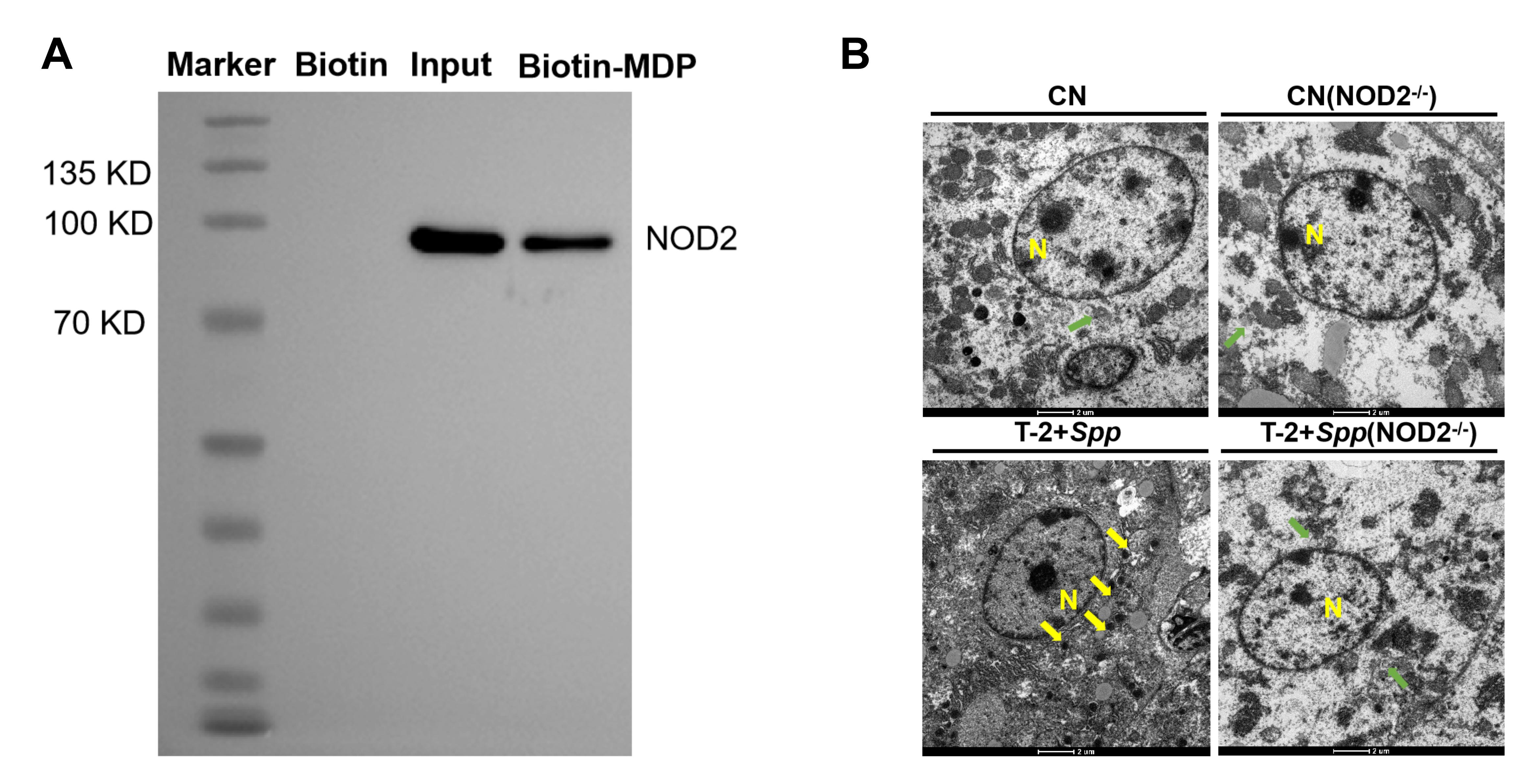


**Figure S3.** NOD2 recognition of S. saprophyticus exacerbates liver immune damage. **A)** Western blot detection results of NOD2 and MDP. **B)** Ultrastructure in the perisinusoidal space revealed by TEM. N means nucleus, green arrow means autophagosome, yellow arrow means autolysosome. Scale bars, 2 μm. *Spp*, *S. saprophyticus*.
